# Chirality of New Drug Approvals (2013–2022): Trends and Perspectives

**DOI:** 10.1021/acs.jmedchem.3c02239

**Published:** 2024-02-12

**Authors:** Rebecca U. McVicker, Niamh M. O’Boyle

**Affiliations:** † School of Pharmacy and Pharmaceutical Sciences, Trinity Biomedical Sciences Institute, Trinity College Dublin, 152−160 Pearse Street, Dublin 2, D02 R590, Ireland; ‡ Gamlen Tableting Ltd, 3 Stanton Way, London SE26 5FU, United Kingdom

## Abstract

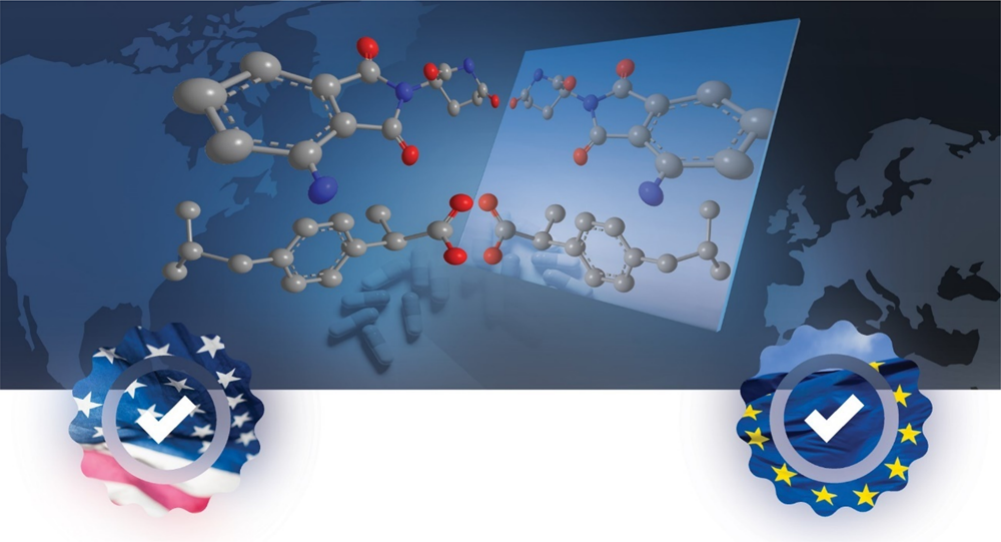

Many drugs are chiral with their chirality determining their biological interactions, safety, and efficacy. Since the 1980s, there has been a regulatory preference to bring single enantiomer to market. This perspective discusses trends related to chirality that have developed in the past decade (2013–2022) of new drug approvals. The EMA has not approved a racemate since 2016, while the average for the FDA is one per year from 2013 to 2022. These 10 include drugs which have been previously marketed elsewhere for several decades, analogues of pre-existing drugs, or drugs where the undefined stereocenter does not play a role in therapeutic activity. Two chiral switches were identified which were both combined with drug repurposing. This combination strategy has the potential to produce therapeutically valuable drugs in a faster time frame. Two class III atropisomers displaying axial chirality were approved between 2013 and 2022, one as a racemate and one as a single enantiomer.

## Significance

- An awareness and understanding of recent trends in new drug approvals has the potential to inform and promote innovation in new drug discovery.
- Making the correct choices regarding drug chirality early in the development process can lead to a substantial cost saving given the cost of the drug approval.
- Determining the impact on patients of practices such as chiral switching and drug repurposing requires data on how often such practices are leveraged.

## Introduction

Since the early 1980s there has been a preference to bring single enantiomer drugs to market over racemates.^1^ In his 1984 paper, E. J. Ariëns stated that ignoring stereoselectivity in the action of drug molecules resulted in “*highly sophisticated scientific nonsense*”.^2^ This rediscovery of the importance of drug stereochemistry combined with new methods of producing enantiomerically pure materials led to a change in regulatory perspectives toward chiral drugs. This eventually led to the publication of the FDA guidance document entitled “Development of New Stereoisomeric Drugs” in 1992 and the EMA guidance document “Investigation of Chiral Active Substances” in 1994.^3,4^

The most common type of molecular chirality results from the presence of one or more stereogenic centers (stereocenters) in a molecule. Carbon atoms are the most common type of stereocenter which gives rise to chirality, although nitrogen, sulfur, and phosphorus stereocenters are not unusual. Chirality does not solely arise from stereogenic atoms. Stereogenic units are also possible. This type of stereochemistry encompasses axial chirality, planar chirality, and helical chirality. Axial chirality arises from the nonplanar arrangement of two pairs of four substituents about an axis.^5,6^ The chiral axis is created by constraints such as steric hindrance or torsional stiffness that prevent free rotation about the axis. This type of chirality is observed in allenes with distinct pairs of substituents and in substituted biaryl compounds where rotation about the aryl–aryl bond is restricted, e.g., BINAP (2,2′-bis(diphenylphosphino)-1,1′-binaphthyl).^6^ This is an example of atropisomerization, where stereoisomers are produced because rotation about a single bond is sufficiently hindered that the barrier to interconversion is high enough to allow separation of the molecules.^6^

Many drugs are chiral with their chirality determining their activity and/or potency. Where one enantiomer is the primary or sole driver for the desired therapeutic effect it is referred to as the eutomer with the other enantiomer being labeled the distomer. Stereochemistry can influence drug target interaction, off-target interactions, absorption, distribution, metabolism, elimination, and excretion.^7,8^ Some differences in the pharmacodynamics and pharmacokinetics are therefore to be expected. In addition to the impact of drug chirality on therapeutic effect, chirality also impacts toxicology, and it is not uncommon for a pair of enantiomers to display dramatically different safety profiles.^8^ Enantiomeric selection during drug development aims to maximize therapeutic effect while minimizing toxicity. It is critical that the possibility of chiral inversion *in vivo* must be considered when establishing the safety profile of a chiral drug. The distomer is generally considered to be an impurity. The eudysmic (or eudismic) ratio is a measure of the activity of the eutomer compared to the distomer in a specified biochemical or biological assay, as the ratio may change depending on the experiment used.^9^ If the enantiomeric purity is not defined, the eudysmic ratio should be interpreted with caution. Single enantiomer drugs are therefore considered to be better defined providing advantages associated with a far higher degree of purity compared to a racemate. Advantages of using an enantiomerically pure drug may include reduced dose requirements, reduced toxicity and side effects, reduced drug interactions, and simpler, better-defined pharmacodynamics and pharmacokinetics.^10^ Making the correct choice during early development can lead to a substantial cost saving given the cost of the drug approval process and the increased costs associated with manufacturing a single enantiomer drug. Chiral HPLC is the most common analytical technique to control enantiomeric purity. Enantiomeric pairs of chiral drug molecules may be classified using the following three categories:^11^

1 One enantiomer is the eutomer, the other the distomer. This is the most common category.
2 The two enantiomers produce the same effect.
3 Chiral inversion occurs *in vivo*. Two types of chiral inversions are possible: unilateral and bilateral. Ibuprofen is an example of a drug that undergoes unilateral inversion *in vivo* while thalidomide undergoes bilateral inversion.

### Examples of Notable Chiral Drugs Approved Pre-2013

**Figure 1:**
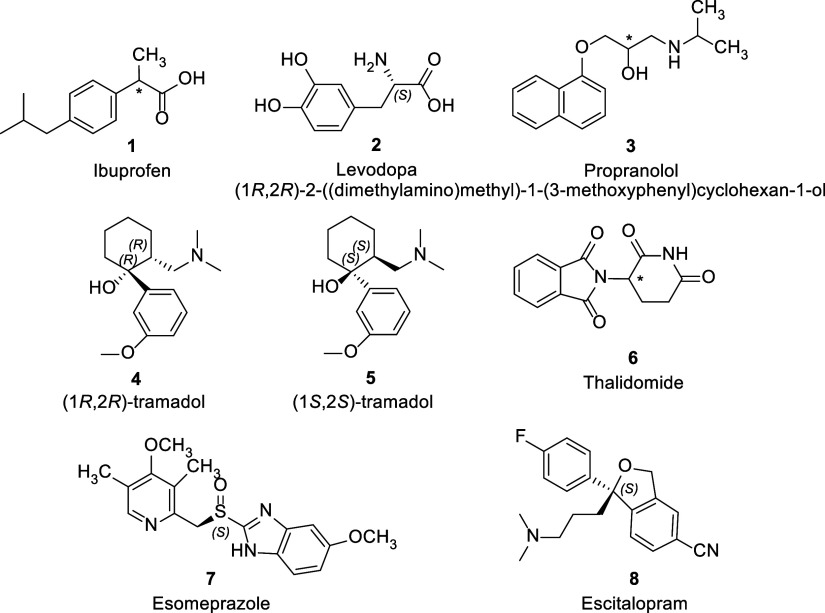
Notable examples of chiral drugs marketed pre-2013 as either racemates or single enantiomers.

**Ibuprofen** is a nonsteroidal anti-inflammatory drug (NSAID) which is usually marketed as a racemate (**1**, Figure 1). Like other 2-arylpropionic acids (ketoprofen, fenprofen, naproxen, etc.), it contains a single stereocenter and the *S*-enantiomer is the eutomer.^10,11^ The eutomer is responsible for the analgesic and anti-inflammatory effect as it displays far greater inhibitory effect on the enzyme cyclooxygenase 1 (COX-1). *In vivo*, approximately 60% of the *R*-enantiomer undergoes unilateral chiral inversion to the *S*-eutomer by a series of enzymatic transformations.^12^ The occurrence of the reverse inversion, eutomer (*S*) to distomer (*R*), is negligible. As such, the *R*-enantiomer acts as a prodrug for the *S*-enantiomer.

**Levodopa**, or l-dopa, is an amino acid precursor to the neurotransmitter dopamine (**2**, Figure 1). It is used in the treatment of Parkinson’s to raise dopamine levels in the central nervous system as it has the ability to cross the blood–brain barrier which dopamine cannot. The distomer, d-dopa cannot be metabolized to dopamine.^13^ The racemate, d/l-dopa, was first investigated for the treatment of Parkinsons in 1967.^14^ It was found to be very effective in the alleviation of symptoms but was associated with unacceptable side effects, including granulocytopaenia. Later investigations where only the eutomer, l-dopa, was administered, resulted in both increased efficacy and reduced toxicity.^15^ This is an example of a chiral drug where the distomer lacks the activity of the eutomer due to differences in how they are metabolized and also causes greater toxicity.

**Propranolol** (**3**, Figure 1) is a competitive, nonselective β-adrenergic receptor antagonist used in the treatment of cardiovascular disorders such as arrhythmia and hypertension. The molecule contains a single stereocenter and is marketed as a racemate. Despite this, like other β-blockers of its class, the desired activity resides in the *S*-enantiomer.^16^ Studies have shown that the *R*-enantiomer does not produce a β-blocking effect, and a half dose of the pure *S*-enantiomer provides the same efficacy as a full dose of the racemate.^17^ Marketing propranolol as a racemate was originally justified on the basis that the distomer did not cause toxic effects, and synthesis of the enantiomerically pure eutomer was especially challenging.^17^ However, claims of increased toxicity related to the *R*-enantiomer have since been made. Several different synthetic routes to the pure eutomer have now been published, but none have been adopted commercially.^18^

**Tramadol** is a chiral analgesic drug which contains two stereocenters giving rise to four possible stereoisomers. It is marketed as a racemic mixture of two of these: (1*R*,2*R*)(+)-tramadol and (1*S*,2*S*)(−)-tramadol (**4** and **5**, Figure 1).^19,20^ Unusually, the enantiomers in the marketed racemate both produce an analgesic effect but by different mechanisms. *In vitro* studies have found that (+)-tramadol shows an affinity for the μ-opioid receptor and inhibits serotonin reuptake, while (−)-tramadol inhibits the reuptake of noradrenaline.^21,22^ Clinical studies have shown that (+)-tramadol produces the best analgesic effect compared to its enantiomer and the racemate, however, it also produces side effects in the form of vomiting and nausea.^20^ The racemate was found to produce a slightly reduced analgesic effect compared to (+)-tramadol. Overall, the racemate produces the most favorable outcome in patients when efficacy and unwanted side effects were considered.

**Thalidomide** (**6**, Figure 1) is easily the most infamous example of a chiral molecule and is regularly used as a cautionary tale when undergraduate students are introduced to stereochemistry. Prescribed to pregnant women in the early 1960s for morning sickness, it interfered with fetal development, causing birth defects.^23^ The devasting effects of this medicine have often been attributed to it being marketed as the racemate as it has been shown that the teratogenic effect resides solely in the *S*(−)-enantiomer. This assertion, however, is incorrect, as the enantiomers have been shown to undergo rapid bilateral interconversion *in vivo*.^8^ Therefore, administration of the racemate or either pure enantiomer has the potential to cause teratogenicity. Since its withdrawal from the market as a treatment for morning sickness, thalidomide has been found to be effective in the treatment of erythema nodosum leprosum, a skin lesion complication associated with leprosy.^10,23^ In addition, it displays immunomodulatory, anti-inflammatory, and antiangiogenic properties making it a useful drug in the treatment of cancer, particularly multiple myeloma.^23^

### Chirality and Drug Approvals

Both EMA and FDA provide guidance on the development of racemates and single enantiomers. The EMA provides guidance on the studies that must be carried out on chiral molecules for marketing authorization applications.^3^ There are four main general categories of marketing authorization applications for new chiral active substance:

(1) Where a single enantiomer is developed as a new active substance, clinical and preclinical studies are only required for the eutomer. However, the possibility of the distomer being formed *in vivo* must be investigated. If it is found to be formed *in vivo* it must be evaluated as a biotransformation product.
(2) Marketing authorization applications for new racemic active substances requires that the choice of the racemate over a single enantiomer be justified. In preclinical studies, pharmacodynamics of the racemate and each enantiomer must be studied and the effective exposure to each enantiomer must be established. Toxicological studies are only required for the racemate unless unpredicted effects are observed at low doses, in which case the individual enantiomers must also be studied. During clinical testing, pharmacodynamic studies are required only on the racemate unless there is a safety requirement to study both enantiomers. Clinical pharmacokinetic studies must employ enantioselective methods unless it has been demonstrated that there is no difference in the fate of the two molecules. Clinical pharmacotherapeutic studies are carried out on the racemate.
(3) The development of a single enantiomer active substance from a previously approved racemate is considered a new application and must be justified. However, data generated for the racemate may be used as part of the application reducing the number of studies required.
(4) Development of a racemate from a single enantiomer is rare and would require justification.

A fifth category referring to development of a nonracemic mixture from an approved racemate or single enantiomer is also mentioned. The FDA requires that the decision to develop a drug as a single enantiomer or a racemate must be justified in the drug approval application.^4^ Their guidance states that stereochemistry should be considered as early as possible in drug discovery projects, and data on each enantiomer should be gathered throughout the development process. Atropisomers are not specifically mentioned by the FDA or EMA in their guidance.

### Chiral Switches

Chiral switching refers to the practice of marketing a single enantiomer of a previously approved racemate or mixture of diastereomers.^24,25^ The justification for this practice is that one enantiomer, the eutomer, provides a greater therapeutic benefit, such as improved efficacy, better bioavailability, or reduced toxicity. Therefore, the single enantiomer is considered to be a more effective drug than the racemate. The definition of a chiral switch may be extended to include the marketing of the opposite enantiomer of a previously approved single enantiomer drug.^24^ In order to be considered a chiral switch, the new drug must differ only in its chirality relative to a previously approved drug. The practice of chiral switching emerged in the 1990s as regulators championed the benefits of enantiomerically pure drugs. In addition to the potential therapeutic advantages, it provided companies with a valuable opportunity to create line extensions for racemic blockbuster drugs and protect against generic intrusion. To this end, chiral switch drugs were preferentially released shortly before the patent of its racemate precursor was due to expire.^26^ In the EMA, a chiral switch drug is considered a new drug approval and so is granted its own period of marketing exclusivity.^3^ In the US, the FDA grants three years of market exclusivity to a chiral switched drug.^25^

A recent review published on the practice of chiral switching has highlighted the limited therapeutic benefits of some chiral switched drugs.^27^ There have also been instances of the development of chiral drugs being halted or being brought to market but later withdrawn due to safety concerns, e.g., dexfenfluramine was withdrawn due to cardiotoxicity and development of (*R*)-fluoxetine was halted due to cardiotoxicity concerns.^28,29^ In addition, a recent meta-analysis comparing clinical trial results of chiral switches with their racemate precursors concluded that the enantiomerically pure drug was “*uncommonly found to provide improved efficacy or safety, despite their greater costs*”.^30^ The FDA does not require preapproval studies comparing the efficacy of chiral switch drugs to the parent racemate.^31^ A study published reviewing chiral switched drugs approved between 2001 and 2011, found that in 6 of the 9 cases, preapproval studies did not include a direct efficacy comparison with racemate.^32^ Where direct comparison was carried out, no evidence of superior efficacy had been demonstrated for the single enantiomer. As such, chiral switching may have an overall negative impact on patients. They must bear the greater cost of the single enantiomer drug while being prevented from accessing generic alternatives without an appreciable therapeutic advantage.

Two well-known examples of chiral switch drugs are esomeprazole and escitalopram. Omeprazole is a proton-pump inhibitor (PPI) used in the treatment of acid-related gastrointestinal disorders such as gastroesophageal reflux disease and peptic ulcer disease. Omeprazole, like other PPIs, inhibits the secretion of acid from gastric parietal cells by irreversibly binding to and inhibiting the activity of H^+^/K^+^ adenosine triphosphatase (ATPase).^33^ It is a chiral compound with a sulfur stereocenter. However, it acts as a prodrug of an achiral active compound. Cleavage of the chiral sulfoxide bond in acidic environments *in vivo* results in the formation of the active sulfonamide. As the first PPI introduced in 1989, omeprazole is considered a “blockbuster” drug, at its peak generating $6.26 billion in sales annually.^34^ It underwent a chiral switch when in 2000, its *S*-enantiomer, esomeprazole (**7**, Figure 1), was brought to market. The justification for this chiral switch was the improved bioavailability of the *S*-enantiomer due to differences in the pharmacokinetic profile of the enantiomers.^35^ Variations in the enzyme CYP2C19 give rise to the presence of fast and slow metabolizers in the population with 3% of Caucasians and 15–20% of Asians being classed as slow metabolizers. The benefit of esomeprazole **7** is a decreased clearance rate dependence on enzyme CYP2C19 such that interindividual pharmacokinetic variation is reduced.^27^ A meta-analysis comparing the clinical effects of the racemate with the pure enantiomer found that more than half of studies found no significant advantage over the racemate and it was noted that most of the studies (9 of 17) employed higher dosages of the single enantiomer.^30^ The chiral switch has, however, provided definite market advantages in the form of patent protection from generic intrusion.^27^

Citalopram is a chiral SSRI (selective serotonin reuptake inhibitor) indicated for the treatment of depression with a single carbon stereocenter. Through the process of chiral switching, the *S*-enantiomer, escitalopram, was brought to market in the US in 2002 (**8**, Figure 1). The *S*-enantiomer is more than 100 times more potent as a serotonin reuptake antagonist compared to the *R*-enantiomer.^36^ This chiral switch has proved to be therapeutically successful. Pooled analysis and meta-analysis of clinical trial data comparing citalopram and escitalopram have supported the therapeutic advantages of escitalopram, including increased potency and reduced dosage requirements.^37,38^ The meta-analysis carried out by Wallach et al. found that all clinical trials either favored the single enantiomer or favored neither despite seven out of eight of the clinical trials employing lower dosages of escitalopram **8**.^30^ The adverse effect profiles of citalopram and escitalopram are similar.^8^

Herein, we analyze and discuss trends related to chirality in the last 10 years of new drug approvals by the FDA and EMA. Data on FDA NME drug approvals was collected from the FDA webpage “New Drugs at FDA: CDER’s New Molecular Entities and New Therapeutic Biological Products”. New drug approvals by the EMA were gathered using “European Public Assessment Reports” (EPARs) and EMA “Human Medicines: Highlights of (year)” reports. Biological drugs were excluded. Full methodological details are described in Supporting Information. Small molecule drug approvals were classified as either racemates, single enantiomers, or achiral entities and further analyzed based on the type and number of stereocenters present. This includes analysis of how often racemates are approved, and the justifications for their approval over the single enantiomer. We also analyzed trends related to chiral switching. As concerns have been raised in the literature that this practice is not advantageous to the patient, it would be beneficial to be aware of how frequently this approach has been leveraged by companies in the past decade.

Agranat et al. have previously published a similar chirality analysis of new drug approvals covering the period 2002–2011 for FDA approvals and 2001–2010 for worldwide approvals.^1^ More recently, Modroiu and Hancu have published an analysis of the chirality of FDA drug approvals during the period 2010–2020.^27^ We extended this analysis to 2021 and 2022 while also reanalyzing FDA drug approvals from 2020 to confirm comparability of the search method employed with previously published data. EMA drug approvals from 2013-2022 were also analysed.

## Results and Discussion

A new molecular entity (NME) is defined by the FDA as a chemical drug that contains no active moiety that has previously been marketed in the USA. This definition excludes biologics. New biologic drugs are referred to as new biologic entities (NBE) by the FDA. The term new therapeutic entities (NTE) encompasses both NMEs and NBEs.^39^ It should be noted that a chiral switch drug may not be considered as a new molecular entity by the FDA as the pure enantiomer was present in the previously marketed racemate.^24^ Further detailed information on FDA approvals from 2020 to 2022 is provided in the Supporting Information (Table S9).

Within the EMA the term new active substance (NAS) is used. This is an all-encompassing term defined as “*a chemical, biological or radiopharmaceutical substance not previously authorised as a medicinal product in the European Union*” or “*an isomer, mixture of isomers, a complex or derivative or salt of a chemical substance previously authorised as a medicinal product in the European Union but differing significantly in properties with regard to safety and efficacy from that chemical substance previously authorised*.”^39^ The term NAS therefore includes biologics. NMEs/NASs were considered. Biopharmaceuticals (biologics) were identified and excluded from this investigation. Also excluded were polymers, as they are not small molecule drugs, and herbal substances, as the active substances of such medicines are poorly defined. Structures of approved racemates (**9**–**25**) are shown in Figure 10 and approved drugs with noncarbon stereocenters^26−29^ are shown in Figure 11. Further detailed information on EMA approvals from 2013 to 2022 is provided in the Supporting Information (Table S10).

### FDA New Drug Approvals Data

#### FDA Biologics and Small Molecule Drug Approvals

**Figure 2:**
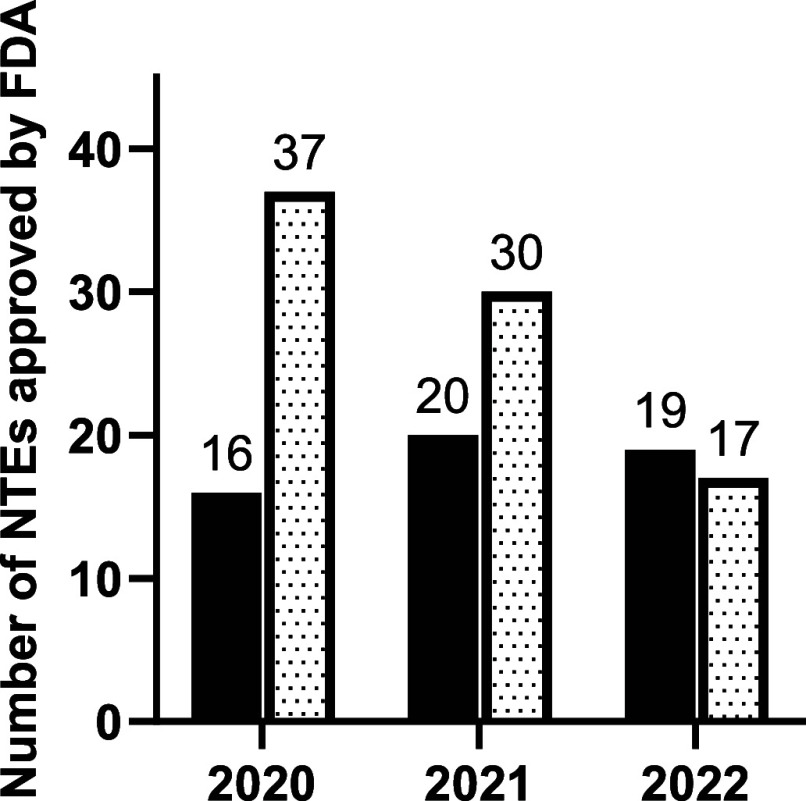
Comparison of the number of biologic (black) and small molecule (white with dots) NTEs approved by the FDA from 2020 to 2022.

Figure 2 and Tables S2 and S9 (Supporting Information) display the FDA NME and NBE approvals data for 2020–2022. The total number of NTEs approved by the FDA’s Centre for Drug Evaluation and Research (CDER) dropped from 53 in 2020 to 36 in 2022. The percentage of these approvals represented by small molecule drugs dropped over this three-year period from 69% to 47%, with the remainder of new approvals comprising biologics. It should be noted that this data, gathered from the FDA Web site, excludes “*vaccines, allergenic products, blood and blood products, plasma derivatives, cellular and gene therapy products, or other products that the Center for Biologics Evaluation and Research approved* ”.^40^ The exclusion of such products reduces the proportion of the biologics.

#### FDA Achiral, Single Enantiomer, and Racemic New Drug Approvals

**Figure 3:**
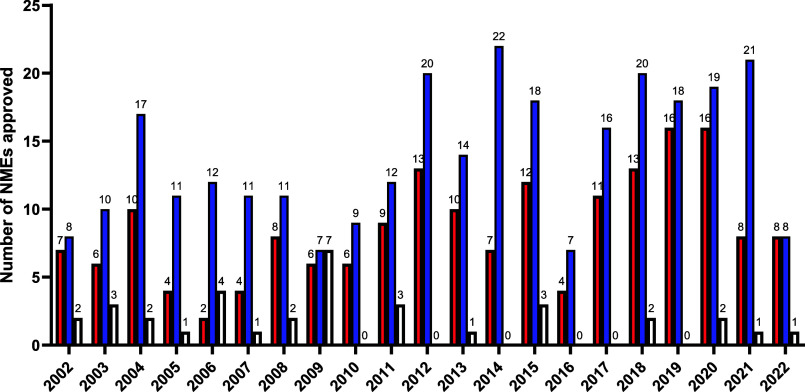
Comparison of the number of achiral (red), single enantiomer (blue), and racemic (white) small molecule NMEs approved by the FDA between 2002 and 2022. Data for years 2020–2022 was gathered by the author. Further data was compiled from ref (27) (years 2010–2020) and ref (1) (years 2002–2011).

Data compiled from the FDA Web site on small molecule NME drug approvals for the years 2020, 2021, and 2022 was classified according to chirality. This data was combined with data from two sources; the paper published by Modroiu and Hancu in 2022 which classified new FDA small molecule drug approvals according to their chirality for the years 2010–2020^27^ and the paper published by Agranat et al. in 2012, which carried out a similar analysis for the years 2002–2011.^1^ The combined data which encompasses the preceding two decades is displayed in Figure 3 and Table S3 (Supporting Information).

In the last 10 years from 2013 to 2022, 10 racemates were approved out of a total of 278 small molecule NME approvals. In the preceding 10 years (2003–2012), 23 out of 211 small molecule NME approvals were racemates. This corresponds to a 3-fold decrease in the percentage of racemic new drug approvals from 11% to 3.6%. Racemic drug approvals are further discussed in the following sections. Comparing the same periods, the percentage of new achiral drug approvals increased from 32% to 38% and new single enantiomer approvals increased from 57% to 59%.

#### Types of Chirality in FDA New Drug Approvals

**Figure 4:**
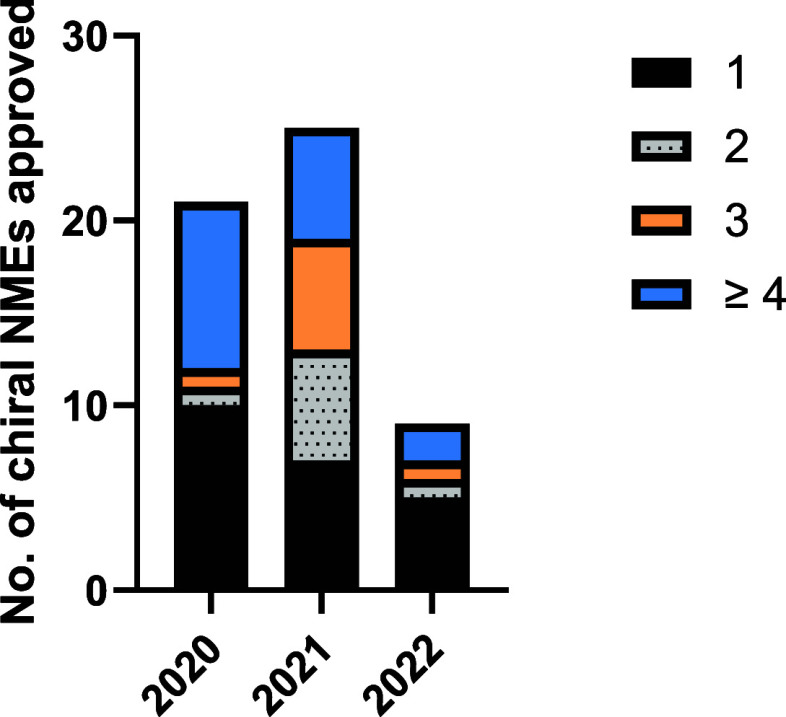
Comparison of the number of stereocenters present in chiral small molecule NMEs approved by the FDA between 2020 and 2022.

All new chiral drugs approved by the FDA in the three years analyzed (2020–2022) contain carbon stereocenters. No other types of chirality, or stereocenter, were identified in NMEs in this period. New chiral drug molecules (both single enantiomers and racemates) were classified according to the number of stereocenters present in the molecule as shown in Figure 4 (and Supporting Information, Table S7). In all three years, the majority of chiral molecules contained a single stereocenter. Molecules containing ≥4 stereocenters represent a substantial proportion of chiral molecules approved each year, with this category representing between 22% and 43% of all chiral approvals across the three years. Additional stereocenters can increase the complexity of chiral drug synthesis as the correct chirality at each stereocenter must be generated and/or maintained throughout the synthesis. Natural product derived drugs and semisynthetic drugs may also have more complex structures with higher numbers of stereocenters. Of the four racemates approved in this period, three contain a single carbon stereocenter (viloxazine, nifurtimox, and amisulpride), and one, gadopiclenol, contains six carbon stereocenter and in marketed as a mixture of diastereomers.

### EMA New Drug Approvals Data

#### EMA Biologics and Small Molecule Drug Approvals

**Figure 5:**
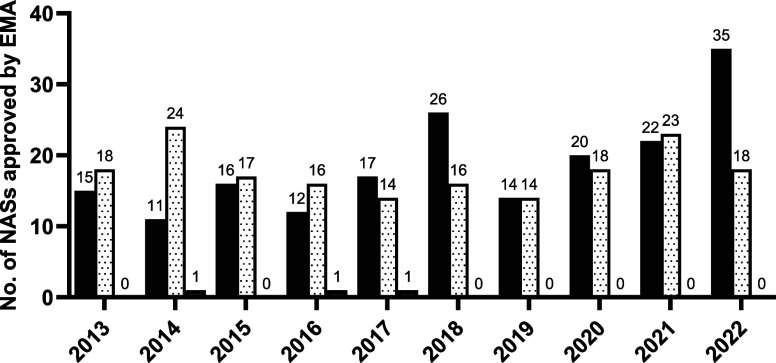
Comparison of the number of biologic (black), small molecule (white with dots), and other (gray) NASs approved by the EMA from 2013 to 2022.

Figure 5 and Table S4 (Supporting Information) display the EMA biologic and small molecule NAS approvals data for the ten-year period from 2013 to 2022. In the first half of the decade analyzed (2013–2017), the EMA approved 163 NASs, an average of 33 per year. This increased to 41 per year in the second half of the decade (2018–2022), totalling 206 for the five-year period. The last three years have generated the highest annual values for the decade while progressively increasing with 2022 reaching 53 NAS approvals.

The proportion of new drug approvals represented by biologic pharmaceuticals has increased over the ten years analyzed. On average, biologics comprised 43% of annual NAS approvals for the first five years of the decade. This increased to an average of 56% in the subsequent five years. In 2022, biologics accounted from 66% of EMA NAS approvals, the highest annual value of the decade. As a result, the proportion of small molecule new drug approvals has decreased. However, the number of annual small molecule new drug approvals has been maintained across the ten-year period. 89 new small molecule drugs were approved in both the first five years and again in the second five years of the decade.

The category of “other” drug approvals encompasses two polymers, tilmanocept and patiromer sorbitex calcium, which were approved in 2014 and 2017, respectively, and one herbal substance, betulae cortex, approved in 2016.

#### EMA Achiral, Single Enantiomer, and Racemic New Drug Approvals

**Figure 6:**
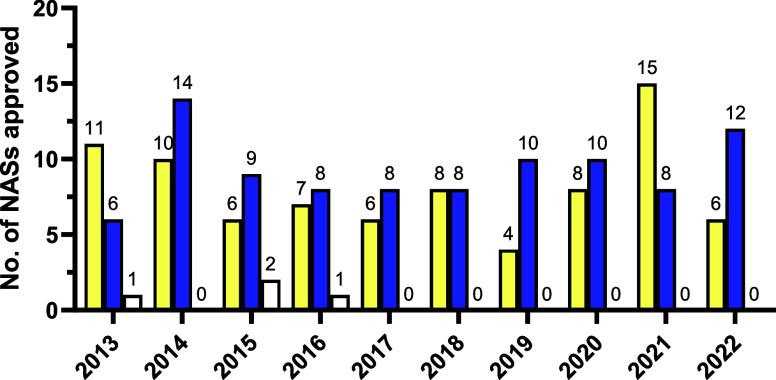
Comparison of the number of achiral (yellow), single enantiomer (blue), and racemic small molecule (white) NAS approved by the EMA from 2013 to 2022.

Figure 6 and Table S5 (Supporting Information) show the results of classifying EMA small molecule NAS approved between 2013 and 2022 according to their chirality. Four racemic NASs were approved by the EMA in the past decade, all prior to 2017. The EMA has not approved any new racemates since lesinurad in 2016. In the five-year period from 2013 to 2017, a total of 40 achiral and 45 single enantiomer small molecule NASs were approved by the EMA. These figures increased slightly for the following five-year period 2018–2022 to 41 and 48, respectively.

#### Types of Chirality in EMA New Drug Approvals

Of the four new racemates approved by the EMA between 2013 and 2022, three contain carbon stereocenters. Two, pomalidomide (**9** and **10**, Figure 10) and panobinostat lactate (**24**, Figure 10) each contain a single carbon stereocenter. Panobinostat lactate was considered a racemic drug for the drug application process, with its chemical name, molecular formula, and relative molecular mass including the lactate moiety. It is notable, however, that the carbon stereocenter that confers its chirality is present in the lactate counterion. EMA and FDA guidelines do not specifically refer to chirality of a counterion, and it is the decision of the manufacturer/applicant how to categorize the application. Isavuconazonium sulfate contains three carbon stereocenters and is marketed as a pair of epimers rather than as a true racemate, i.e., as a pair of diastereomers that differ in stereochemistry at a single stereocenter (**25**, Figure 10). The fourth racemate approved in this period, lesinurad, does not contain any stereocenters but exhibits axial chirality (**11** and **12**, Figure 10). It is marketed as a 50:50 mixture of two atropisomers.

The chirality of the vast majority of single enantiomer small molecule NASs approved in the ten-year period studied arises from the presence of carbon stereocenters. Three single enantiomer drug molecules [sofosbuvir (**26**), tenofovir alafenamide (**27**), and remdesivir (**28**, Figure 11)] contained a single phosphorus stereocenter in addition to other carbon stereocenters. Avibactam (**29**, Figure 11) was the only small molecule NAS approved in this period to contain a nitrogen stereocenter. Two carbon stereocenters are also present in the avibactam molecule.

**Figure 7:**
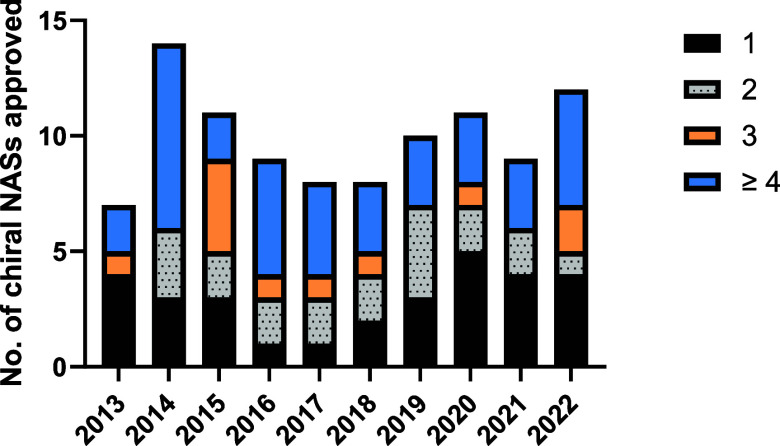
Comparison of the number of stereocenters present in chiral small molecule NASs approved by the EMA between 2013 and 2022.

Thirty of the 97 new chiral active substances approved by the EMA in the past decade contain a single stereocenter. Thirty-six contain ≥4 stereocenters as shown in Figure 7 and Table S8 (Supporting Information). No clear trend in the number of stereocenters present in EMA small molecule NAS approvals is discernible over this period.

#### EMA Drug Type Analyzed by Therapeutic Area

**Figure 8:**
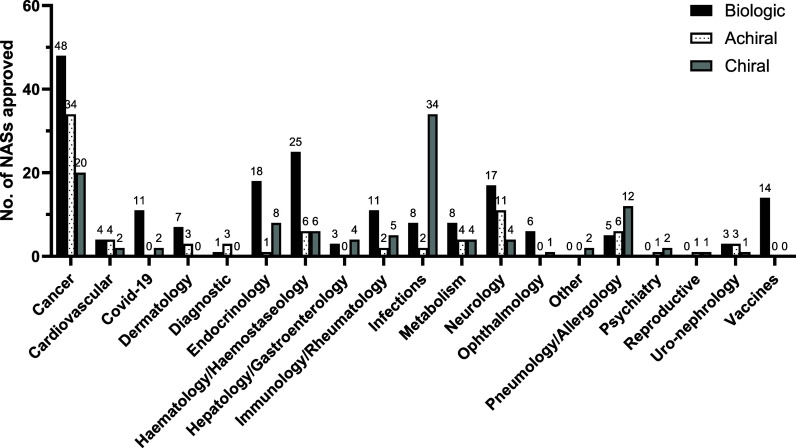
Categorization of biologic (black), achiral (white with dots), and chiral (gray) NASs approved by the EMA between 2013 and 2022 according to the general therapeutic area for which they are indicated.

The EMA “Human Medicines: Highlights of (year)” reports classified new drug approvals according to the general therapeutic area in which they are approved for use. As these reports were not published prior to 2015, new EMA drug approvals for the years 2013 and 2014 were classified using the same therapeutic area categories by the authors. In Figure 8 and Table S6 (Supporting Information), EMA NAS drug approvals from 2013 to 2022 are categorized by their respective therapeutic areas and classified as either biologic, achiral, or chiral drugs. This provides an insight into the medical areas in which the different types of NAS approvals are more prevalent.

Small molecule chiral drugs have dominated the NAS approvals for the treatment of infections for the last 10 years. 34 new chiral active substances were approved for the treatment of infections accounting for 77% of NASs approved in this area. This includes isavuconazonium sulfate, which is classed as a racemate as it is marketed as a mixture of two epimers (**25**, Figure 10). Approximately one-third of drugs within this category were approved for the treatment of HIV and another third for the treatment of hepatitis C. This is an area which has traditionally seen success in drug discovery from chiral natural products or semisynthetic derivatives. Several approvals between 2013 and 2022 follow this trend, e.g., the semisynthetic glycopeptide oritavancin. Several reviews have been published on the importance of stereochemistry in the mechanism of different classes of antivirals.^41−44^ In their 2023 paper, Chibale et al. argue that that reducing the cost of drugs used in the treatment of infectious diseases is critical to tackling these diseases and preventing them spreading.^45^ Single enantiomer drugs are inherently more expensive to produce compared to racemates. Therefore, they argue that where a new racemate does not display unacceptable toxicity, the racemate should be preferentially marketed over a single enantiomer. Currently, many existing antimalarial drugs are marketed as racemates.^46^

The majority of NASs approved for pneumology/allergology (52%), hepatology/gastroenterology (57%), and psychiatry (2 out of 3) were chiral. A substantial proportion of NASs approved for the following categories were also chiral: endocrinology (30%), immunology/rheumatology (28%, including one racemic drug), metabolism (25%), and reproductive (1 out of 2). Chiral drugs represented only 20% of NASs for the treatment of cancer. However, as the largest therapeutic category this represents 20 chiral NAS approvals. This includes the two racemic drugs, pomalidomide (**9** and **10**, Figure 10) and panobinostat lactate (**24**, Figure 10).

### Comparison of EMA and FDA Data

**Figure 9:**
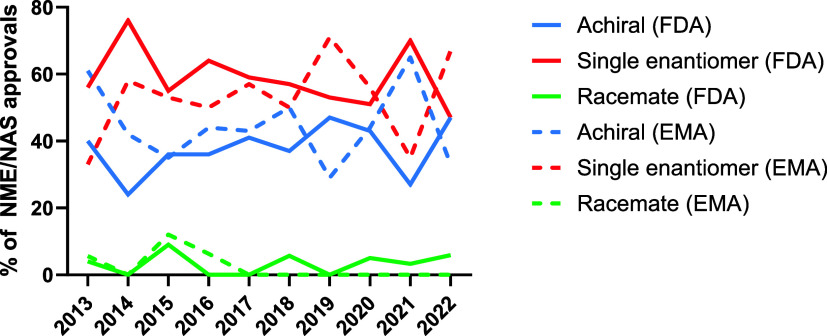
Comparison of the percentage of achiral, single enantiomer, and racemic NMEs/NASs (excluding biologics) approved by the FDA and EMA from 2013 to 2022.

Figure 9 displays achiral, single enantiomer, and racemic small molecule NME/NAS approvals for the 10 years of FDA and EMA drug approvals from 2013 to 2022, expressed as a percentage of total small molecule NME/NAS approvals (excluding biologics). As previously noted, the proportion of FDA small molecule drug approvals represented by racemates decreased significantly between the past decade (2013–2022) and the preceding decade (2003–2012). However, examination of trends within the past decade show that racemic drugs represent a similar proportion of FDA small molecule drug approvals in the second half of the decade compared to the first. In the five years from 2013 to 2017, racemates accounted for 3.2% of FDA small molecule drug approvals, while in the subsequent five years they account for 3.9%. By contrast, there is a downward trend for EMA racemic drug approvals. In the first half of the decade, racemates accounted for 4.5% of new EMA small molecule drug approvals, whereas in the second half of the decade, no new racemic active substances were approved.

Variations in the proportions of new small molecule drug approvals represented by achiral and single enantiomer drugs were minimal for both the FDA and the EMA. In the first half of the decade (2013–2017), achiral drugs represented 35% of FDA new small molecule drug approvals, increasing to 40% in the second half of the decade (2018–2022). The proportion of single enantiomer drugs approved dropped from 62% to 56%. Comparing the same time periods for EMA small molecule drug approvals, achiral drugs accounted for 45% of approvals and single enantiomer drugs for 51% in the first five-year period (2013–2017). These values increased to 46% (achiral drugs) and 54% (single enantiomer drugs) in the second five-year period (2018–2022).

### Racemic Drugs Approved by the EMA and FDA between 2013 and 2022

Considering the preference for single enantiomer drugs, data related to racemic drug approvals were further examined to assess reasons for bringing a racemate to market. All racemic small molecule NMEs/NASs approved in the ten-year period from 2013 to 2022 are listed in Table 1 (FDA) and Table 2 (EMA). Three true racemates were approved by the FDA between 2020 and 2022 containing one carbon stereocenter each. The fourth “racemate” approved in this period is a mixture of diastereomers containing six undefined carbon stereocenters.

**Table 1:** List of All Racemic NMEs Approved by The FDA from 2013 to 2022

year approved	medicine name	active substance	therapeutic area	indication/use	no. stereocenters
2013	Pomalyst	pomalidomide	cancer	multiple myeloma	1
2015	Zurampic	lesinurad	rheumatology	gout	0^a^
2015	Farydak	panobinostat lactate	cancer	multiple myeloma	1^b^
2015	Cresemba	isavuconazonium sulfate	infections	aspergillosis	3^c^
2018	Diacomit	stiripentol	neurology	anticonvulsant	1
2018	Krintafel	tafenoquine	infection	malaria	1
2020	Barhemsys	amisulpride	gastroenterology	nausea and vomiting	1
2020	Lampit	nifurtimox	infection	Chagas disease	1
2021	Qelbree	viloxazine	psychiatry	ADHD	1
2022	Elucirem	gadopiclenol	diagnostic imaging	detection and visualization of lesions	6^d^

a Lesinurad displays axial chirality.

b The stereocenter is located on the lactate counterion.

c One of the three stereocenters of isavuconazonium sulfate is undefined. It is marketed as a mixture of epimers.

d The stereochemistry of all six stereocenters of gadopiclenol is undefined. It is marketed as a mixture of diastereomers.

**Table 2:** List of All Racemic NASs Approved by the EMA from 2013 to 2022

year approved	medicine name	active substance	therapeutic area	indication/use	no. stereocenters
2013	Imnovid	pomalidomide	cancer	multiple myeloma	1
2015	Farydak	panobinostat lactate	cancer	multiple myeloma	1^a^
2015	Cresemba	isavuconazonium sulfate	infections	aspergillosis	3^b^
2016	Zurampic	lesinurad	rheumatology	gout	0^c^

a The stereocenter is located on the lactate counterion.

b One of the three stereocenters of isavuconazonium sulfate is undefined. It is marketed as a mixture of epimers.

c Lesinurad displays axial chirality.

#### Pomalidomide

**Figure 10:**
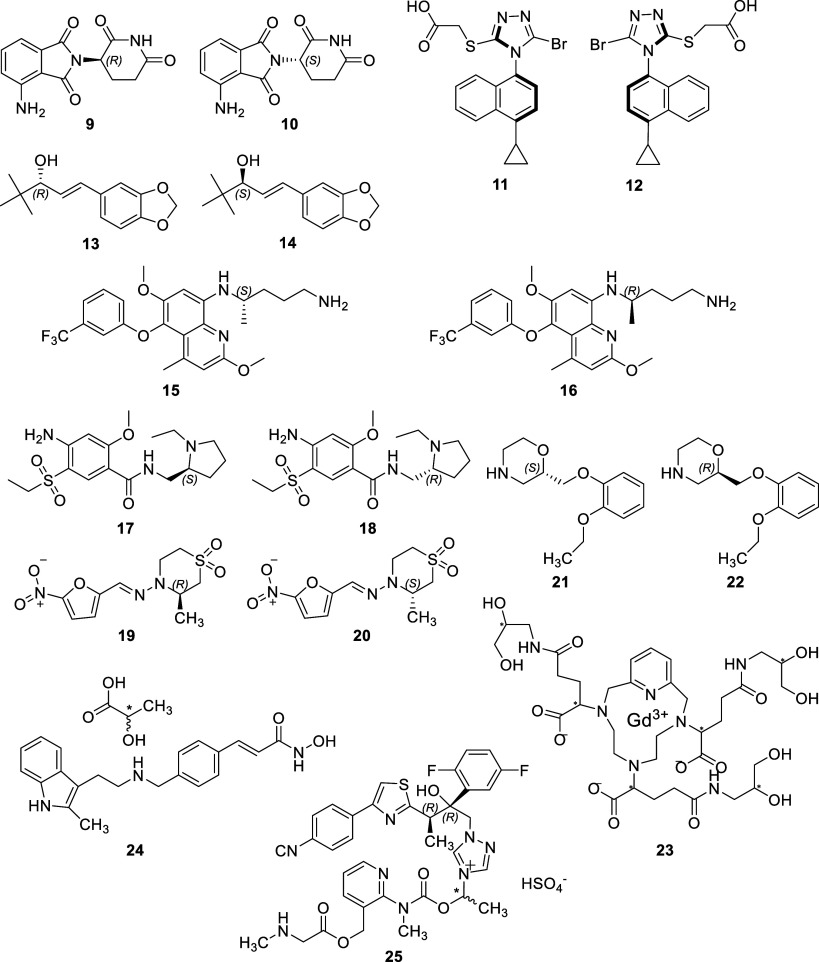
Racemic or diastereomeric drugs approved by the FDA and/or EMA in the period 2013–2022.

Pomalidomide was approved by the FDA in 2013 as Pomalyst and by the EMA in the same year as Imnovid for the treatment of multiple myeloma in patients whose disease progressed after being treated with at least two other cancer drugs. It is an analogue of thalidomide that exhibits immunomodulatory, antiproliferative, and antiangiogenic activity.^47^ Like thalidomide, it contains a single carbon stereocenter and its enantiomers readily undergo bilateral interconversion *in vivo*, hence the decision to market it as a racemate (**9** and **10**, Figure 10).

**Figure 11:**
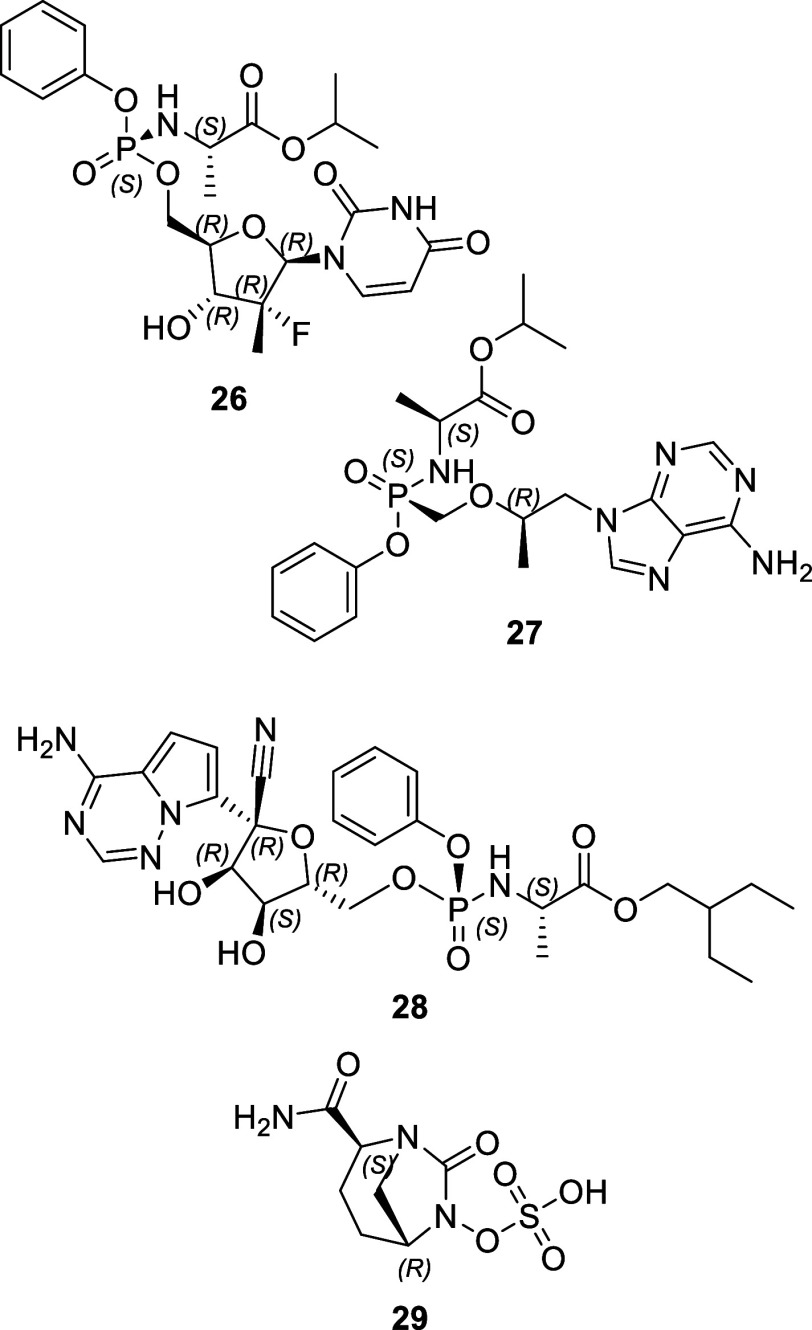
Drug approvals (2013–2022) containing a noncarbon stereocenter.

#### Lesinurad

Lesinurad was marketed for the treatment of hyperuricaemia in patients with gout as Zurampic by the FDA in 2015 and the EMA in 2016. It has since been withdrawn from both markets at the request of the market authorization holder for business reasons.^48^ The therapeutic benefit of lesinurad arises from its inhibition of transporter proteins responsible for uric acid reabsorption in the kidneys.^49^ Lesinurad does not contain any stereocenters; instead, it displays a less common form of chirality known as axial chirality (**11** and **12**, Figure 10).^50^ Axial enantiomers are atropisomers, i.e., conformational isomers in which rotation about a single bond is sufficiently hindered such that separation of the enantiomers is possible. The stability of atropisomers varies greatly depending on the level of hindrance to rotation such that the half-life for racemization can vary from an order of seconds to years.^51^ A study published in 2017 separated and assessed the atropisomers of lesinurad.^50^ It was found that each atropisomer was stable, with no interconversion being observed. It was also hypothesized that (−)-lesinurad may be a more effective treatment for hyperuricaemia compared to the racemate. It displayed improved activity for the inhibition of the transfer protein, hURAT1, and more favorable pharmacokinetics.

#### Stiripentol

In 2018, stiripentol was approved as Diacomit by the FDA for the treatment of seizures in children with Dravet syndrome, a form of epilepsy. Diacomit had previously been granted market authorization in the EU in 2007. Stiripentol is marketed as a racemate with both enantiomers displaying the desired anticonvulsant activity (**13** and **14**, Figure 10). Stiripentol produces its desired therapeutic effect through multiple mechanisms. It acts as an allosteric modulator of GABA_A_ receptors inhibiting the uptake of GABA (γ-aminobutyric acid).^52^ It also inhibits the metabolism of other anticonvulsant drugs when administered concurrently. The *R*(+)-enantiomer **13** was found to be 2.4 times more potent than the *S*(−)-enantiomer **14** with the potency of the racemate falling between that of the two enantiomers.^53^ However, **13** is eliminated faster as it has a shorter half-life and a higher rate of plasma clearance compared to **14**.

#### Tafenoquine

Tafenoquine was approved as Krintafel for the treatment and prevention of malaria caused by *Plasmodium vivax* and *Plasmodium ovale* by the FDA in 2018. It is classed as an 8-aminoquinoline and is a long-acting analogue of another antimalarial, primaquine. Tafenoquine is effective as a single dose, whereas primaquine requires a two-week treatment.^54^ Like several other antimalarials, such as chloroquine, hydroxychloroquine, mefloquine, and halofantrine, tafenoquine contains a single carbon stereocenter and is marketed as a racemate (**15** and **16**, Figure 10).^46^ Primaquine and tafenoquine are the only available treatments which are active against both the liver hypnozoites and the sexual blood stages of malaria.^55^ The difference in plasma concentrations between the two tafenoquine enantiomers in human trials was found to be less than 10%.^56^ Enantiomers of 8-aminoquinolines have been shown to differ in the terms of their antimalarial activity.^55,57^

#### Amisulpride

The racemic drug, amisulpride (**17** and **18**, Figure 10), was granted marketing authorization under the brand name Barhemsys in 2020 by the FDA for the prevention of nausea and vomiting after surgery. Amisulpride is also an atypical antipsychotic and has been approved for the treatment of psychiatric conditions outside of the US for over 30 years.^58^ The therapeutic benefits of amisulpride have previously been solely attributed to its activity as a selective dopamine D_2_ and D_3_ receptor antagonist. However, more recently, it has been shown to also be serotonin 5-HT_7_ receptor antagonist.^59^ The *S*(−)-enantiomer **17** displays a 40-fold increase in potency for the D_2_ receptor compared to the *R*(+)-enantiomer **18**, while **18** displays a 50-fold potency increase for the 5-HT_7_ receptor.^60^ The racemic form of amisulpride therefore provides a polypharmaceutical therapeutic advantage over the individual enantiomers.

#### Nifurtimox

Nifurtimox was originally introduced in 1965 for the treatment of Chagas’ disease. Although it had previously been possible to obtain nifurtimox in the US directly through the CDC, it was not granted marketing authorization by the FDA until 2020.^61^ Nifurtimox is associated with high levels of toxicity and side effects, including mutagenicity; however, it is one of only two drugs currently available of the treatment of Chagas’ disease. Chagas’ disease is caused by the protozoan parasite, *Trypanosoma cruzi*. Nifurtimox acts to reduce the presence of the parasite in the blood, thereby reducing the likelihood of chronic complications and death.^62^ A study published in 2015 concluded that it was unlikely that a single enantiomer of nifurtimox would have a therapeutic advantage over the racemate (**19** and **20**, Figure 10).^63^ This conclusion was based on an observed lack of stereoselectivity in the toxicity, pharmacokinetics, and activity of nifurtimox against *T. cruzi*.

#### Viloxazine

Viloxazine was first approved for the treatment of depression in the UK in 1974. It was available in several European countries for the same indication until the early 2000s, when it was withdrawn from the market for business reasons.^64^ Following repurposing as an ADHD treatment, it was introduced to the US market for the first time in 2021 as Qelbree (**21** and **22**, Figure 10). The therapeutic effect of viloxazine derives from its action as a selective norepinephrine reuptake inhibitor.^65^ There is also evidence that it may impact the dopamine and serotonin systems of the brain. Comparison of the enantiomers of viloxazine have shown that the *S*(−)-enantiomer **21** is five times more active for the desired therapeutic effect compared to the *R*(+)-enantiomer **22**.^65,66^

#### Gadopiclenol

Gadopiclenol (**23**, Figure 10) is a macrocyclic, gadolinium-based contrast agent (GBCA) used to detect and visualize lesions with abnormal vascularity in combination with MRI (magnetic resonance imaging). Gadopiclenol produces a large magnetic moment when placed in magnetic field.^67^ This in turn creates a local magnetic field, enhancing the relaxation rate of water molecules in the vicinity. As a result, the MRI signal intensity is enhanced in the effected tissues. Gadopiclenol was first approved under the trade name Elucirem by the FDA in 2022 as a diagnostic imaging agent. In 2006, a link between renal toxicity and GBCAs was identified. Further investigation established that this toxicity was only associated with linear and not macrocyclic GBCAs due to their reduced kinetic stability.^68^ This led to the development of the paramagnetic, macrocyclic, nonionic complex of gadolinium, gadopiclenol (**23**, Figure 10). The gadopiclenol molecule contains six carbon stereocenters such that 2^6^ = 64 diastereomers can be present in solution, although there is an element of symmetry in the molecule.^68^ Gadopiclenol is a DOTA (dodecane tetraacetic acid or tetraxetan) complex. As in the case of godopiclenol, the kinetic inertness of such complexes can be increased by adding substituents so that steric bulk and chirality are introduced, which in turn increases the rigidity of the ligand backbone.^69^

#### Panobinostat Lactate

Panobinostat is a nonselective histone deacetylase inhibitor indicated for the treatment of multiple myeloma.^70^ It is sold as Farydak in the lactate anhydrous form. Farydak was granted marketing authorization by the FDA and the EMA in 2015 but was withdrawn from the US market in 2022. The FDA approval of Farydak had been accelerated and had included a requirement for a postmarketing trial in order to confirm the drug’s therapeutic benefit.^71^ The market authorization holder, Secura Bio, submitted a request to the FDA to have market authorization withdrawn, as it was not feasible to carry out the required clinical trials. The panobinostat free base is achiral. However, racemic lactic acid is used to generate the lactate counterion (**24**, Figure 10).^72^

#### Isavuconazonium Sulfate

Isavuconazonium sulfate (**25**, Figure 10) is indicated for the treatment of aspergillosis. It acts as a prodrug, being rapidly hydrolyzed *in vivo* to the antifungal, isavuconazole.^73^ It is sold under the brand name Cresemba and was granted marketing approval by the FDA and EMA in 2015. The isavuconazonium molecule contains three carbon stereocenters, two of which reside in the active isavuconazole moiety and are defined.^74^ Isavuconazonium is racemic with respect to the third stereocenter, which resides in the inactive cleavage product. As such isavuconazonium is composed of a pair of epimers, diastereomers that differ in chirality at a single stereocenter, as opposed to a true racemate.

### Chiral Switch Data

We were further interested in assessing trends in the practice of chiral switching. Table 3 lists new active substances approved by the FDA and EMA that have arisen from a chiral switch strategy between 1976 and 2022 compiled from refs (26, 27, 30, 75, and 76). In the ten years from 2013 to 2022, no NASs approved by the EMA were identified as chiral switches. In the same period, two new active substances arising from chiral switches were identified in the FDA new drug approvals, namely levomilnacipran and esketamine.

**Table 3:** List of Chiral Switch Drugs and Their Parent Racemates^a^

racemic drug		single enantiomer drug			
name	year approved in USA	name	enantiomer	year approved (region)	pharmacological activity or indication^d^
albuterol	1981	levabuterol	(*R*)(−)-albuterol	1999 (USA)	β_2_ adrenergic receptor agonist antiasthmatic
amphetamine	1960	dextroamphetamine	(*S*)(+)-amphetamine	1976 (USA)	stimulant for treatment of ADHD and narcolepsy
betaxolol	1985	levobetaxolol	(*S*)(−)betaxolol	2000 (USA)	β_1_ adrenergic receptor antagonist for hypertension and elevated intraocular pressure
bupivacaine	1972	levobupivacaine	(*S*)(−)-bupivacaine	1999 (USA)	local anesthetic
cetirizine	1995	levocetirizine	(*R*)(−)-cetirizine	2001 (Europe); 2007 (USA)	H_1_ antihistamine
citalopram	1998	escitalopram	(*S*)(+)-citalopram	2001 (Europe); 2002 (USA)	SSRI antidepressant
fenfluramine	1973/2020^b^	dexfenfluramine	(*S*)(+)-fenfluramine	1996 (USA)^c^	antiobesity
formoterol	2001	arformoterol	(*R*,*R*)(−)-formoterol	2006 (USA)	β_2_ adrenergic receptor agonist antiasthmatic, COPD
ibuprofen	1974	dexibuprofen	(*S*)(+)-ibuprofen	1994 (Austria)	nonsteroidal anti-inflammatory (NSAID)
ketamine	1970	esketamine	(*S*)(+)-ketamine	2019 (USA); 1997 (Germany)	general anesthetic/antidepressant
ketoprofen	1986	dexketoprofen	(*S*)(+)-ketoprofen	1998 (Europe)	nonsteroidal anti-inflammatory (NSAID)
lansoprazole	1995	dexlansoprazole	(*R*)(+)-lansoprazole	2009 (USA)	PPI antacid
leucovorin	1952	levoleucovorin	(*S*)(−)-leucovorin	2008 (USA)	folate deficiency, treatment of colorectal carcinoma, decreases toxic effects of methotrexate and pyrimethamine
methylphenidate	1995	dexmethylphenidate	(*R*,*R*)(+)-methylphenidate	2001 (USA)	stimulant for treatment of ADHD and narcolepsy
milnacipran	2009	levomilnacipran	(*S*,*R*)(−)-milnacipran	2013 (USA)	SNRI antidepressant
modafinil	1998	armodafinil	(*R*)(−)-modafinil	2007 (USA)	narcolepsy treatment
ofloxacin	1980	levofloxacin	(*S*)(−)-ofloxacin	1996 (USA); 1997 (Europe)	antibacterial
omeprazole	1989	esomeprazole	(*S*)(−)-omeprazole	2000 (Europe); 2001 (USA)	PPI antacid
zopiclone	1986	eszopiclone	(*S*)(+)-zopiclone	2004 (USA)	hypnotic sedative for anxiety and insomnia

a Compiled from refs (26, 27, 30, 75, 76).

b Racemic fenfluramine was withdrawn from the market in 2015. It has since been repurposed and reintroduced to the market in 2020 as an antiseizure drug.

c Dexfenfluramine was withdrawn from the market in 1997.

d ADHD = attention deficit hyperactivity disorder, SSRI = selective serotonin reuptake inhibitor, COPD = chronic obstructive pulmonary disease, PPI = proton-pump inhibitor, SNRI = serotonin–norepinephrine reuptake inhibitor.

**Figure 12:**
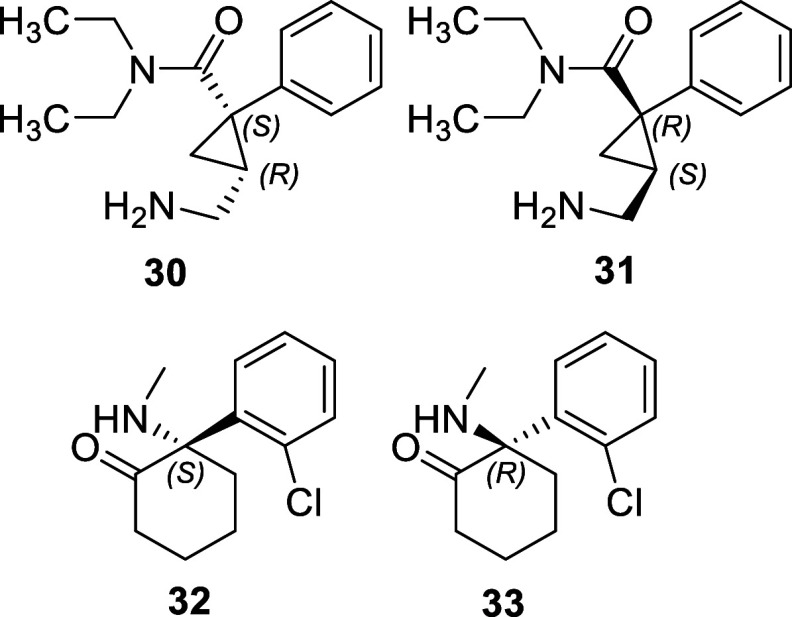
Two recent examples of chiral switching. (1*S*,2*R*)(−)-milnacipran (levomilnacipran) **30** and (1*R*,2*S*)(+)-milnacipran **31**, esketamine **32**, and arketamine **33**.

Milnacipran is a serotonin–norepinephrine reuptake inhibitor (SNRI). Racemic (1*R*,2*S*/1*S*,2*R*)(±)-milnacipran (**30** and **31**, Figure 12) was approved for the treatment of fibromyalgia in adults by the FDA in 2009 under the brand name Savella. In 2013, the FDA approved the medicine Fetzima, containing the single (1*S*,2*R*)(−)-enantiomer, levomilnacipran (**30**, Figure 12), as the active ingredient for the treatment of major depressive disorder. This is therefore an example of drug repurposing being combined with a chiral switch strategy. Levomilnacipran **30** had previously been marketed within the EU for the treatment of major depressive disorder since 1996.^76^ However, in 2009, the EMA refused to grant marketing approval to a (±)-milnacipran product indicated for the treatment of fibromyalgia.^77^ The reasons provided for the refusal included a lack of evidence to support efficacy or maintenance of effect.

A 2013 paper compared the activity of levomilnacipran with its enantiomer **31** (Figure 12) and the racemate, (±)-milnacipran.^78^ Levomilnacipran **30** exhibited affinities at least 10 times higher than its enantiomer for rat and human norepinephrine (NETs) and serotonin (SERTs) transporters. It was also found to be a 50 times more potent inhibitor of norepinephrine reuptake and a 13 times more potent inhibitor serotonin reuptake in rat hypothalamic synaptosomes compared to its enantiomer. The authors concluded that levomilnacipran **30** was the active enantiomer in terms of SNRI activity. In addition, it was found to have a more favorable pharmacokinetic profile.

Ketamine has been marketed as a general anesthetic under the brand name Ketalar in the US since it was granted approval by the FDA in 1970.^76^ It is widely used as a general anesthetic and is included on the World Health Organisation (WHO) List of Essential Medicines for this use. Its *S*(−)-enantiomer, esketamine (**32**, Figure 12), has been marketed in several countries, outside the US, as a general anesthetic since the 1990s. In 2019, esketamine was granted its first FDA approval as Spravato, a nasal spray for the treatment of treatment resistant depression (TRD) in adults. This FDA approval therefore represents both a chiral switch and an example of drug repurposing. Spravato was also approved by the EMA for TRD treatment in 2019. As **32** was already marketed in the EU, this is not an instance of chiral switching within the EMA but is considered drug repurposing.

The anesthetic effect of ketamine arises from its activity as a NMDA (*N*-methyl-d-aspartate) receptor antagonist.^79^ It has been found that esketamine **32** exhibits a 3-fold increase in anesthetic potency compared to the *R*-enantiomer arketamine **33** in humans.^80^ Ketamine has been known to bind to a number of other receptors including opioid, nonopioid sigma, muscarinic, and serotonin receptors which have been proposed as the basis of its analgesic effect.^79^ Ketamine also generates several metabolites which have been implicated in its therapeutic activity, including norketamine and hydroxynorketamine.^81,82^ The chiral sense of the parent drug enantiomer is retained in both of these metabolites.

The mechanism of action responsible for the antidepressant activity of ketamine is unique compared to existing antidepressants, which are typically SSRIs or SNRIs. The onset of antidepressant effects for ketamine are rapid, within 2 h, compared to several weeks for SSRIs or SNRIs, and sustained, lasting approximately 7 days.^79^ It has also been found to reduce suicidal ideation. The antidepressant mechanism of ketamine has not been definitively established but appears complex, with mechanisms both related and unrelated to its activity as an NMDA receptor agonist being implicated.^79^ In preclinical trials in rodents, the *R*(+)-enantiomer, arketamine (**33**, Figure 12), was found to display superior antidepressant activity compared to the *S*-enantiomer **32** or the racemate.^83^ In addition, it produced the lowest level of side effects. An open-label pilot study was carried out to investigate the antidepressant activity of arketemine **33** in 2021.^84^ Results showed a substantial improvement in patient mood within 24 h of an intravenous dose of **33**.

### Racemic Drug Approvals

In 2012, Agranat et al. published a paper entitled “The predicated demise of racemic new molecular entities is an exaggeration”.^1^ This statement appears to be upheld by the list of 10 new racemic active substances approved by the FDA and/or EMA between 2013 and 2022. However, it should be taken into consideration that several of these active substances are not entirely new. Stiripentol, amisulpride, nifurtimox, and viloxazine have all been in use for several decades in regions outside of the US but only recently granted approval by the FDA. Pomalidomide (**9** and **10**, Figure 10) and tafenoquine (**15** and **16**, Figure 10) are analogues of pre-existing drugs. Of the remaining four drugs, the undefined stereocenters of two of them, panobinostat lactate (**24**, Figure 10) and isavuconazonium sulfate (**25**, Figure 10), reside outside the active moiety of the drug. The two remaining drugs are lesinurad, which does not display conventional stereocenter based chirality but instead axial chirality, and gadopiclenol, which is marketed as a mixture of many diastereomers. As noted previously, lesinurad was subsequently withdrawn from both markets for business reasons. There have been no truly novel racemic drugs, in which an undefined stereocenter plays a role in therapeutic activity, approved by the FDA or EMA in the past decade. For several decades, regulatory agencies have been clear in their preference for bringing single enantiomer drugs to market over racemates. However, where an undefined stereocenter does not play a role in therapeutic activity of the drugs or where the drug, or its analogue, has been marketed elsewhere for an extended period, marketing of the racemate appears to be more accepted to regulators.

### The Future of Chiral Switching

Similarly, the two drugs resulting from chiral switches approved by the FDA in the past decade, levomilnacipran and esketamine, had previously been marketed outside the US since the 1990s. Interestingly, in both cases, the single enantiomer drug was indicated for a different use compared to the parent racemate. This suggests that the practice of developing chiral switch drugs for the purposes of line extensions is dying out. Instead, the practice of combining chiral switching with drug repurposing is developing as a new trend.

Fenfluramine also underwent a chiral switch combined with repurposing within the past decade. Racemic fenfluramine was originally marketed as an appetite suppressant in the short term treatment of obesity. It underwent an initial chiral switch and the *S*-enantiomer, dexfenfluramine, was brought to market for the long-term treatment of obesity. Both were withdrawn by the FDA in 1990s due to evidence of cardiotoxicity, resulting in valvular heart disease.^29^ Finlepta, containing racemic fenfluramine, is a treatment for seizures associated with Dravet syndrome that was approved by the FDA and EMA in 2020.^76^ Finlepta is therefore a result of drug repurposing and a chiral switch back to the racemate. Finlepta is not listed in the above examples of chiral switch drugs, as *rac*-fenfluramine was previously marketed in the regions of interest and therefore not a new active substance. Preclinical testing in zebrafish has indicated that the (+)-enantiomer of fenfluramine has a greater antiseizure activity compared to the opposite enantiomer.^85^ As such, the future may hold yet another chiral switch for fenfluramine.

Drug repurposing refers to the practice of “*identifying new uses for approved or investigational drugs that are outside the scope of the original medical indication*”.^86^ The benefit of this strategy in that the time and cost required for the drug to reach the market is reduced as the discovery and early development phases are bypassed and existing data on side effects, pharmacodynamics in humans, etc., can be utilized. This strategy is of particular importance in the search for drugs to treat rare diseases where there is less incentive for companies to invest in drug discovery and development. The benefits of combining chiral switch and drug repurposing strategies include improved drug safety and/or efficacy, reduced development expenses, faster approval time, higher likelihood of a marketing exclusivity period, and patentability.^76^

Moreover, marketing a chiral switch drug for a different indication to the parent drug circumvents the concerns that have been raised in the literature regarding the therapeutic benefits of chiral switch drugs. As previously noted, several authors have raised concerns regarding the lack of evidence supporting the claims that the new single enantiomer drugs, derived from marketed racemates, display a superior therapeutic benefit.^27,30,87^ The benefits of evergreening or line extension of the parent product are certain, however, these business benefits do not apply in the case of a repurposed drug. It would be of interest to see a review of currently marketed racemic drugs that have been shown in preclinical and/or clinical tests to have enantiomers that display markedly different activities. Such information on a variety of racemic drugs would inform future chiral switch/drug repurposing combination strategies.

Another facet of chiral switching is the development of deuterium-enabled chiral switch drugs. This concept involves swapping a hydrogen atom substituent of a stereocenter with a deuterium atom. This stabilizes the stereocenter through the deuterium kinetic isotope effect, thus reducing the possibility of enantiomer/diastereomer inversion.^88^ This is of particular use in the case of chiral molecules that undergo enantiomer inversion *in vivo*, such as thalidomide and its analogues. DeWitt et al. have successfully utilized this strategy to separate and investigate the activities of individual enantiomers of several thalidomide analogues including lenalidomide and avadomide. In each case, they found that the eutomer displayed markedly superior antitumorigenic properties compared to the distomer.^88,89^ To date, no new drugs have been brought to market using this strategy.

Given that fewer racemic drugs are being brought to market, the opportunity to market a single enantiomer of a previously approved racemate is reducing. However, chiral switching can also refer to the practice of marketing the opposite enantiomer of a previously approved single enantiomer. This type of chiral switch may become more prevalent within the context of drug repurposing. Given the reported improved antidepressant activity of arketamine relative to esketamine or the racemate, such a chiral switch to the opposite enantiomer may provide a therapeutic benefit.^83,84^

Nonetheless, opportunities for the classic chiral switch approach still exist. For instance, there is evidence in the literature that there would be a therapeutic advantage to marketing viloxazine and lesinurad, two racemic drugs approved in the last 10 years, as single enantiomers.^50,65,66^ The key to avoiding misuse of this strategy for economic rather than therapeutic gain is the inclusion of direct comparisons of the single enantiomer and the racemate in the marketing authorization application. However, this is not currently required by the FDA or EMA.^31,90^ The cost of producing a single enantiomer drug is also a consideration.

### Atropisomerism and Axial Chirality

Atropisomers are conformational isomers where rotation about a single bond is sufficiently hindered to allow separation. This can create a pair of enantiomers or diastereomers displaying axial chirality. LaPlante categorized molecules with a suitable atropisomeric axis according to the rate of axial rotation about that bond.^91^ Where the half-life of conversion (*t*_1/2_) is in the order of seconds or faster, a pair of molecules are not considered to be atropisomers and do not exhibit axial chirality (class I). The *t*_1/2_ of class II compounds falls between 60 s and 4.5 years, and class III compounds display *t*_1/2_ greater than 4.5 years. Class II and class III compounds are considered atropisomers and can exhibit axial chirality.

A limitation of our search strategy is that axial chirality arising from atropisomerism has the potential to be overlooked, specifically in the case of class II atropisomers. Stable class III atropisomers are expected to be clearly identified, and class I molecules are not considered chiral. Atropisomerism has become more prevalent in pharmaceutical compounds in recent years. This has been linked to the increased use of aromatic heterocycles as functional groups.^51^ A recent analysis found that approximately 30% of small molecule drugs approved between 2010 and 2018 fall into class I.^92^ A total of four class III drugs have ever been approved by the FDA, including the racemic drug described above, lesinurad.^51^ One of these drugs, sotorasib, was also approved within the past decade but marketed as a single enantiomer. Sotorasib is indicated for the treatment of nonsmall cell lung cancer. The decision to market as a single enantiomer was based on the observed 10-fold difference in potency between the atropisomers.^93^

The key difference compared to classical stereocenter derived chirality is that racemization of atropisomers does not require bond breaking but only bond rotation. Atropisomerism has been described as a “lurking menace” in relation to drug discovery.^91^ This is of particular concern for class II compounds. Given the time scale of racemization for these compounds, stability issues could easily occur within the production, quality testing, or patient administration timeframes. For this reason, class II compounds are rarely brought to market. Instead, several strategies have been developed that may be leveraged during drug discovery to circumnavigate this issue when a class II compound has shown desirable therapeutic benefits. These include introducing symmetry into the molecule to eliminate chirality, engineering faster bond rotation to eliminate atropisomerism or increasing steric hindrance about the axis to further stabilize the atropisomers.^91^

Recently, rather than approach atropisomersim as a difficulty to be overcome, it has been used as a key component of new drug design.^51^ Considering atropisomerism in combination with both chiral switching and drug repurposing, an approach to drug discovery is proposed. There is an abundance of class I compounds on the market, e.g., lenacapavir. The separate activities of the rotamers of these compounds are not typically investigated due to difficulties in isolating them. Introduction of steric hindrance about their axial bonds would create a pair of enantiomers which are analogues of the original compound. Investigating the activities of these new molecules could lead to improved therapeutic activity for the original indication or possibly for different indications. Where considerable differences exist between the enantiomers, this could inform further drug discovery. In their paper published in 2023, Gillis et al. utilized this strategy as part of their discovery of new antiretroviral drugs for the treatment of HIV.^94^ They produced analogues of the existing HIV drug lenacapavir which exhibits atropisomerism. Steric hindrance around the aryl–aryl bond increased the stability of the analogues allowing the separation of the individual atropisomers.

## Conclusions

Racemic drug approvals have not entirely died out in the past decade (2013–2022). However, 6 out of 10 of the new racemates approved by the FDA and/or EMA in this time were either marketed for several decades elsewhere or are analogues of well-known drugs. None of the remaining four contain an undefined stereocenter which plays a role in therapeutic activity. Novel drugs are no longer being brought to market which contain clinically relevant undefined stereocenters. This finding emphasizes the importance of stereoselective synthetic approaches and characterization techniques within the current pharmaceutical manufacturing landscape. Yet, the possibility of marketing new drugs as racemates should not been ignored. As Chibale et al. assert, the cost benefit to the patient of marketing a racemate should be considered where the safety profile of the racemate is acceptable.^45^ Moreover, there are instances where the racemate produces an improved therapeutic effect because of the combined action of enantiomers, e.g., tramadol and stiripentol.

The classic chiral switch approach has disappeared in the past decade. Overall, this is considered a positive development given the lack of evidence that it has been beneficial to the patient. A new trend has developed combining chiral switching with drug repurposing.^76^ This combination strategy provides many advantages and avoids the downfalls of the classic chiral switch approach. Currently, only two drugs have been brought to market using this strategy. Further exploitation of this approach has the potential to produce therapeutically valuable drugs within a condensed time frame.

Axial chirality, arising from atropisomerism, should become a greater topic of focus in drug discovery. Two of the four class III compounds authorized by the FDA were approved in the past decade. A review has found that 26% of small molecule drugs approved by the FDA in the period 2018 to early 2022 contain an atropisomeric axis.^51^ This form of chirality is more difficult to identify and less well-known compared to stereocenter-based chirality. It has the potential to be a powerful drug design tool but also to disrupt drug development programs when atropisomerism is unidentified. As such, axial chirality merits further investigation and greater attention in drug discovery.

Overall, our findings provide an insight into the trends that have developed with regard to the chirality of FDA and EMA new small molecule drug approvals in the last 10 years. Leveraged correctly, they have the potential inform and stimulate future drug discovery, design, and development. On the basis of our investigations, it would be advantageous to update the current FDA and EMA guidelines from the early 1990s to include guidance on, for example, chirality in counterions and atropisomerism.

## Supporting Information Available

Methods for compiling EMA new drug approvals data; methods for compiling FDA new drugs approvals data; methods for identifying chiral switches (PDF)
Searchable data table of FDA NTE Classification (2020–2022); searchable data table of EMA NAS Classification (2013–2022) (XLSX)

## Abbreviations Used

5-HT: 5-hydroxytryptamine
ADHD: attention deficit hyperactivity disorder
BINAP: 2,2′-bis(diphenylphosphino)-1,1′-binaphthyl
DOTA: dodecane tetraacetic acid
EMA: European Medicines Agency
EPAR: European Public Assessment Report
GBCA: gadolinium-based contrast agent
NAS: new active substance
NBE: new biologic entity
NCE: new chemical entity
NET: norepinephrine transporter
NTE: new therapeutic entity
PPI: proton-pump inhibitor
SNRI: serotonin–norepinephrine reuptake inhibitor
TRD: treatment resistant depression
WHO: World Health Organisation
