# Supplementary material for: Chirality of New Drug Approvals (2013–2022): Trends and Perspectives
Source: J Med Chem. 2024 Feb 12;67(4):2305–20. doi: 10.1021/acs.jmedchem.3c02239 (PMC10895675; doi:10.1021/acs.jmedchem.3c02239)
Supplement: Supplementary file 1 — jm3c02239_si_001.pdf [file jm3c02239_si_001.pdf]

***Supporting Information: jm-2023-02239b***

## Chirality of New Drug Approvals (2013-2022): Trends and Perspectives

**Rebecca U. McVicker[1,2] and Niamh M. O'Boyle[1*]**

[1]School of Pharmacy and Pharmaceutical Sciences, Trinity Biomedical Sciences Institute, Trinity College Dublin, 152 - 160 Pearse St, Dublin 2, D02 R590 Ireland

[2]Gamlen Tableting Ltd, 3 Stanton Way, London SE26 5FU, United Kingdom

**Corresponding Author Information:** Email: nioboyle@tcd.ie

## Table of Contents

Methods ..... S1
Compiling EMA New Drug Approvals Data ..... S2
*Table S1. Naming conventions for selected groups of biological drugs.* ..... S3
Compiling FDA New Drug Approvals Data ..... S4
Compiling Chiral Switch Data ..... S4
Search Strategy Limitations ..... S4
Table S2. Comparison of the number and percentage of biologic and small molecule NTEs approved by the FDA from 2020-2022. ..... S5
Table S3. Comparison of the number and percentage of achiral, single enantiomer and racemic NMEs approved by the FDA between 2002-2022. ..... S6
Table S4. Comparison of the number and percentage of biologic and small molecule NASs approved by the EMA from 2013-2022. ..... S7
Table S5. Comparison of the number and percentage of achiral, single enantiomer and racemic small molecule NASs approved by the EMA from 2013-2022. ..... S8
Table S6. Categorisation of biologic, achiral and chiral NASs approved by the EMA between 2013-2022 according to the general therapeutic area for which they are indicated. ..... S9
Table S7. Comparison of the number of stereocentres present in chiral small molecule NMEs approved by the FDA between 2020-2022. ..... S10
Table S8. Comparison of the number of stereocentres present in chiral small molecule NASs approved by the EMA between 2013-2022. ..... S10
FDA NTE Classification (2020-2022) ..... S11
*Table S9. All medicines containing new therapeutic entities (NTEs) approved for marketing authorisation by the FDA from 2020-2022 classified as either biologic, achiral, single enantiomer or racemate.* ..... S11
EMA NAS Classification (2013-2022) ..... S20
*Table S10. All medicines containing new active substances (NASs) approved for marketing authorisation by the EMA from 2013-2022 classified as either biologic, achiral, single enantiomer, racemate, polymer or herbal substance.* ..... S20
References ..... S44

# Methods

Terminology is used in accordance with IUPAC (International Union of Pure and Applied Chemistry) definitions stated in "Basic Terminology of Stereochemistry".(1)

## COMPILING EMA NEW DRUG APPROVALS DATA

The EMA "Human Medicines: highlights of (year)" reports were used to identify medicines which contained a NAS which were approved by the EMA in the years 2015 to 2022.(2) These reports provide the trade names of new medicines which have been approved each year and classify them according to their general therapeutic area. Those which contain a NAS are highlighted. No other information on the drug is provided. Based on these reports a list was compiled of the trade names of all medicines approved by the EMA in the period 2015-2022 which contained a NAS.

This list was then cross-referenced with a spreadsheet listing all medicine European Public Assessment Reports (EPARs) downloaded from the EMA website.(3) This spreadsheet provides significantly more detail than the highlights reports including medicine name, active substance(s), exact marketing authorisation date, use/indication etc. It does not, however, indicate which medicines contain active substances which have not previously been authorised thus necessitating the use of the "Human Medicines: highlights of (year)" reports.

The final stage in compiling the EMA data was classification of the NASs according to their chirality. The list of active substances was firstly filtered to remove the majority of biologics. This was achieved by removing drugs classed as vaccines then leveraging naming conventions used for biopharmaceuticals. NASs whose name contained the suffixes listed in

**Table S1** were classified as biologics and not considered for further classification. Similarly, all vaccines were classed as biologics and not further classified.

The remaining medicines were then classified according to their chirality; chiral or achiral. Some additional biologics and non-biologic macromolecules were identified which were excluded from the chirality classification. Chiral molecules were further classified according to whether they are marketed as a single enantiomer or as a racemate. For simplicity, mixtures of diastereomers were classified as racemic drugs. Chiral drug molecules were also classified based on their type of chirality and, in the case of molecules containing one or more stereocentres, the number and stereocentre type. This was achieved by accessing the EPARs on the EMA website for each medicine. The Active Substance section of each report provides the structure of the active substance and details of the molecule's chirality.

As the EMA "Human Medicines: highlights of (year)" reports were not published prior to 2015, an alternative approach was employed to the compilation of EMA data for years 2013 and 2014. The list of all medicines approved in those years from the Medicine EPAR spreadsheet was filtered to remove biologics. The EPARs for all remaining medicines were accessed on the EMA website. These reports were used to identify which active substances were NASs and also classify them according to their chirality as described above.

***Table S1. Naming conventions for selected groups of biological drugs.*** *(4)*

| **Biological Drug Group** | **Suffix** |
|---|---|
| Antisense oligonucleotides | -rsen |
| Cell therapies | -cel |
| Enzymes | -ase |
| Gene therapies | -gene |
| Gene therapy vectors | -vec |
| Monoclonal antibodies | -mab |
| Peptides and glycopeptides | -tide |

## COMPILING FDA NEW DRUG APPROVALS DATA

Data on FDA NME drug approvals was collected from the FDA web page "New Drugs at FDA: CDER's New Molecular Entities and New Therapeutic Biological Products".(5) This site lists the trade names of all medicines approved by the FDA since 2015 that contain NMEs or NBEs. In addition, it provides the name of the new active substance, approval date and the medicine's approved use. Biologics were filtered from the extracted list based on their nomenclature as described above (**Table S1**). The remaining medicines were cross-referenced with the EMA list such that the classifications of medicines also appearing in the EMA list could be transposed. In order to classify the remaining NMEs according to their chirality, their structures were obtained from PubChem, the website of the US National Library of Medicine.(6) The stereochemistry of each compound was confirmed using the Global Substance Registration System website.(7)

As data has previously been published by Modroiu and Hancu classifying FDA drug approvals from 2010-2020 based on their chirality, only new FDA drug approvals from 2020-2022 were analysed as part of the study.(8) 2020 was included in this analysis to confirm that the previously published data was replicated using the approach described here.

## COMPILING CHIRAL SWITCH DATA

In order to identify new active substances that had been brought to market in the last ten years using the chiral swich approach, the naming conventions for chiral switch drugs was leveraged. The prefixes *es*- and *ar*- may be used where a racemate is chiral switched to the *S* or *R* enantiomer, respectively. Alternatively, the prefixes *dextro*- or *dex*- may be used for a chiral swich drug that displays dextrorotary optical activity or *levo*- or *lev*- where is displays levorotary optical activity.(9) The EPARs spreadsheet from the EMA website was filtered for the relevant time period (2013-2022).(3) Searches for the chiral switch prefixes were carried out within the filtered list. Each search result was examined to determine whether it was the result of chiral switch within the period of interest. Several active substances fulfilled the search criteria but were not the result of a chiral switch strategy e.g. artesunate, aripiprazole, estetrol monohydrate. Others were the result of chiral switching but had previously been marketed prior to 2013 e.g. esomeprazole, levofloxacin.

The same search procedure was applied to the compiled list of FDA drug approvals from 2020-2022. For the years 2013-2019, this search procedure was applied to the relevant lists of new drug approvals on the FDA web page "New Drugs at FDA: CDER's New Molecular Entities and New Therapeutic Biological Products".(5)

## SEARCH STRATEGY LIMITATIONS

A limitation of this search strategy is that axial chirality arising from atropisomerism has the potential to be overlooked, specifically in the case of Class II atropisomers. Stable Class III atropisomers are expected to be clearly identified and Class I molecules are not considered chiral.

**Table S2. Comparison of the number and percentage of biologic and small molecule NTEs approved by the FDA from 2020-2022.**

| **Year** | **Biologic** | | **Small Molecule** | | **Total No.** |
|---|---|---|---|---|---|
| | **No.** | **%** | **No.** | **%** | |
| 2020 | 16 | 31 | 37 | 69 | 53 |
| 2021 | 20 | 39 | 30 | 61 | 50 |
| 2022 | 19 | 53 | 17 | 47 | 36 |
| Total 2020-2022 | 55 | 40 | 84 | 60 | 139 |

**Table S3. Comparison of the number and percentage of achiral, single enantiomer and racemic NMEs approved by the FDA between 2002-2022.**

Data for years 2020-2022 was gathered by the author. Further data was compiled from reference (8) (years 2010-2020) and reference (10) (years 2002-2011).

| Year | Achiral | | Single Enantiomer | | Racemate | | Total No. |
|---|---|---|---|---|---|---|---|
| | No. | % | No. | % | No. | % | |
| 2002 | 7 | 41 | 8 | 47 | 2 | 12 | 17 |
| 2003 | 6 | 32 | 10 | 53 | 3 | 16 | 19 |
| 2004 | 10 | 34 | 17 | 59 | 2 | 6.9 | 29 |
| 2005 | 4 | 25 | 11 | 69 | 1 | 6.3 | 16 |
| 2006 | 2 | 11 | 12 | 67 | 4 | 22 | 18 |
| 2007 | 4 | 25 | 11 | 69 | 1 | 6.3 | 16 |
| 2008 | 8 | 38 | 11 | 52 | 2 | 10 | 21 |
| 2009 | 6 | 30 | 7 | 35 | 7 | 35 | 20 |
| 2010 | 6 | 40 | 9 | 60 | 0 | 0.0 | 15 |
| 2011 | 9 | 38 | 12 | 50 | 3 | 13 | 24 |
| 2012 | 13 | 39 | 20 | 61 | 0 | 0.0 | 33 |
| 2013 | 10 | 40 | 14 | 56 | 1 | 4.0 | 25 |
| 2014 | 7 | 24 | 22 | 76 | 0 | 0.0 | 29 |
| 2015 | 12 | 36 | 18 | 55 | 3 | 9.0 | 33 |
| 2016 | 4 | 36 | 7 | 64 | 0 | 0.0 | 11 |
| 2017 | 11 | 41 | 16 | 59 | 0 | 0.0 | 27 |
| 2018 | 13 | 37 | 20 | 57 | 2 | 5.7 | 35 |
| 2019 | 16 | 47 | 18 | 53 | 0 | 0.0 | 34 |
| 2020 | 16 | 43 | 19 | 51 | 2 | 5.0 | 37 |
| 2021 | 8 | 27 | 21 | 70 | 1 | 3.3 | 30 |
| 2022 | 8 | 47 | 8 | 47 | 1 | 5.9 | 17 |
| Total 2003-2012 | 68 | 32 | 120 | 57 | 23 | 11 | 211 |
| Total 2013-2022 | 105 | 38 | 163 | 59 | 10 | 3.6 | 278 |
| Total 2002-2022 | 180 | 36 | 291 | 58 | 35 | 6.9 | 506 |

**Table S4. Comparison of the number and percentage of biologic and small molecule NASs approved by the EMA from 2013-2022.**

| Year | Biologic | | Small Molecule | | Other | | Total No. |
|---|---|---|---|---|---|---|---|
| | No. | % | No. | % | No. | % | |
| 2013 | 15 | 45 | 18 | 55 | 0 | 0.0 | 33 |
| 2014 | 11 | 31 | 24 | 67 | 1 | 2.8 | 36 |
| 2015 | 16 | 48 | 17 | 52 | 0 | 0.0 | 33 |
| 2016 | 12 | 41 | 16 | 55 | 1 | 3.4 | 29 |
| 2017 | 17 | 53 | 14 | 44 | 1 | 3.1 | 32 |
| 2018 | 26 | 62 | 16 | 38 | 0 | 0.0 | 42 |
| 2019 | 14 | 50 | 14 | 50 | 0 | 0.0 | 28 |
| 2020 | 20 | 53 | 18 | 47 | 0 | 0.0 | 38 |
| 2021 | 22 | 49 | 23 | 51 | 0 | 0.0 | 45 |
| 2022 | 35 | 66 | 18 | 34 | 0 | 0.0 | 53 |
| Total 2013-2017 | 71 | 44 | 89 | 55 | 3 | 1.8 | 163 |
| Total 2018-2022 | 117 | 57 | 89 | 43 | 0 | 0.0 | 206 |
| Total 2013-2022 | 188 | 51 | 178 | 48 | 3 | 0.8 | 369 |

**Table S5. Comparison of the number and percentage of achiral, single enantiomer and racemic small molecule NASs approved by the EMA from 2013-2022.**

| Year | Achiral | | Single Enantiomer | | Racemate | | Total No. |
|---|---|---|---|---|---|---|---|
| | No. | % | No. | % | No. | % | |
| 2013 | 11 | 61 | 6 | 33 | 1 | 5.6 | 18 |
| 2014 | 10 | 42 | 14 | 58 | 0 | 0.0 | 24 |
| 2015 | 6 | 35 | 9 | 53 | 2 | 12 | 17 |
| 2016 | 7 | 44 | 8 | 50 | 1 | 6.3 | 16 |
| 2017 | 6 | 43 | 8 | 57 | 0 | 0.0 | 14 |
| 2018 | 8 | 50 | 8 | 50 | 0 | 0.0 | 16 |
| 2019 | 4 | 29 | 10 | 71 | 0 | 0.0 | 14 |
| 2020 | 8 | 44 | 10 | 56 | 0 | 0.0 | 18 |
| 2021 | 15 | 65 | 8 | 35 | 0 | 0.0 | 23 |
| 2022 | 6 | 33 | 12 | 67 | 0 | 0.0 | 18 |
| Total 2013-2017 | 40 | 45 | 45 | 51 | 4 | 4.5 | 89 |
| Total 2018-2022 | 41 | 46 | 48 | 54 | 0 | 0.0 | 89 |
| Total 2013-2022 | 81 | 46 | 93 | 52 | 4 | 2.2 | 178 |

**Table S6. Categorisation of biologic, achiral and chiral NASs approved by the EMA between 2013-2022 according to the general therapeutic area for which they are indicated.**

| Therapeutic Area | Biologic % | Achiral % | Chiral % | Other % |
|---|---|---|---|---|
| Cancer | 47 | 33 | 20 | 0 |
| Cardiovascular | 40 | 40 | 20 | 0 |
| Covid-19 | 85 | 0 | 15 | 0 |
| Dermatology | 64 | 27 | 0 | 9 |
| Diagnostic agents | 20 | 60 | 0 | 20 |
| Endocrinology | 67 | 4 | 30 | 0 |
| Haematology/ Haemostaseology | 68 | 16 | 16 | 0 |
| Hepatology/ Gastroenterology | 43 | 0 | 57 | 0 |
| Immunology/ Rheumatology | 61 | 11 | 28 | 0 |
| Infections | 18 | 5 | 77 | 0 |
| Metabolism | 50 | 25 | 25 | 0 |
| Neurology | 53 | 34 | 13 | 0 |
| Ophthalmology | 86 | 0 | 14 | 0 |
| Other | 0 | 0 | 100 | 0 |
| Pneumology/ Allergology | 22 | 26 | 52 | 0 |
| Psychiatry | 0 | 33 | 67 | 0 |
| Reproductive | 0 | 50 | 50 | 0 |
| Uro-nephrology | 38 | 38 | 13 | 13 |
| Vaccines | 100 | 0 | 0 | 0 |

**Table S7. Comparison of the number of stereocentres present in chiral small molecule NMEs approved by the FDA between 2020-2022.**

| Year | No. of NMEs with X No. of Stereocentres | | | | | | | | | | | |
|---|---|---|---|---|---|---|---|---|---|---|---|---|
| | **1** | **2** | **3** | **4** | **5** | **6** | **7** | **8** | **9** | **10** | **11** | **12** |
| 2020 | 10 | 1 | 1 | 2 | 0 | 4 | 1 | 1 | 0 | 1 | 0 | 0 |
| 2021 | 7 | 6 | 3 | 3 | 0 | 0 | 1 | 0 | 0 | 0 | 0 | 2 |
| 2022 | 5 | 1 | 1 | 0 | 0 | 1 | 0 | 1 | 0 | 0 | 0 | 0 |

**Table S8. Comparison of the number of stereocentres present in chiral small molecule NASs approved by the EMA between 2013-2022.**

| Year | No. of NAS with X No. of Stereocentres | | | | | | | | | | | |
|---|---|---|---|---|---|---|---|---|---|---|---|---|
| | **1** | **2** | **3** | **4** | **5** | **6** | **7** | **8** | **9** | **10** | **11** | **12** |
| 2013 | 4 | 0 | 1 | 1 | 1 | 0 | 0 | 0 | 0 | 0 | 0 | 0 |
| 2014 | 3 | 3 | 0 | 1 | 3 | 4 | 0 | 0 | 0 | 0 | 0 | 0 |
| 2015 | 3 | 2 | 4 | 1 | 1 | 0 | 0 | 0 | 0 | 0 | 0 | 0 |
| 2016 | 1 | 2 | 1 | 1 | 1 | 1 | 1 | 0 | 1 | 0 | 0 | 0 |
| 2017 | 1 | 2 | 1 | 1 | 0 | 0 | 1 | 2 | 0 | 0 | 0 | 0 |
| 2018 | 2 | 2 | 1 | 1 | 1 | 1 | 0 | 0 | 0 | 0 | 0 | 0 |
| 2019 | 3 | 4 | 0 | 2 | 1 | 0 | 0 | 0 | 0 | 0 | 0 | 0 |
| 2020 | 5 | 2 | 1 | 0 | 0 | 1 | 0 | 0 | 0 | 0 | 1 | 0 |
| 2021 | 4 | 2 | 0 | 0 | 0 | 0 | 1 | 1 | 0 | 0 | 0 | 0 |
| 2022 | 4 | 1 | 2 | 2 | 0 | 2 | 0 | 0 | 0 | 0 | 0 | 1 |

# FDA NTE Classification (2020-2022)

*Table S9. All medicines containing new therapeutic entities (NTEs) approved for marketing authorisation by the FDA from 2020-2022 classified as either biologic, achiral, single enantiomer or racemate.*

*Where more than one active substance is listed, the NTE(s) is shown in bold. Where a medicine contains more than one NTE, an additional entry has been added for each additional NTE. Data was gathered from the FDA website and excludes "vaccines, allergenic products, blood and blood products, plasma derivatives, cellular and gene therapy products, or other products that the Center for Biologics Evaluation and Research approved".(5)*

*Note: an Excel file containing these tables is also available as Supporting Information.*

| Medicine Name | Specific Therapeutic Area | Active Substance | Marketing Authorisation Date | Drug Type/ Chirality | No. Stereo-centres | Type of Stereo-centres |
|---|---|---|---|---|---|---|
| **NexoBrid** | To remove eschar in adults with deep partial thickness or full thickness thermal burns | anacaulase-bcdb | 28/12/2022 | Biologic | - | - |
| **Briumvi** | To treat relapsing forms of multiple sclerosis | ublituximab-xiiy | 28/12/2022 | Biologic | - | - |
| **Xenoview** | To evaluate pulmonary function and imaging | hyperpolarized Xe-129 | 23/12/2022 | Achiral | - | - |
| **Lunsumio** | To treat adults with relapsed or refractory follicular lymphoma, a type of non-Hodgkin lymphoma | mosunetuzumab-axgb | 22/12/2022 | Biologic | - | - |
| **Sunlenca** | To treat adults with HIV whose HIV infections cannot be successfully treated with other available treatments due to resistance, intolerance, or safety considerationsPress Release | lenacapavir | 22/12/2022 | Single Enantiomer | 3 | C |
| **Krazati** | To treat KRAS G12C-mutated locally advanced or metastatic non-small cell lung cancer in adults who have received at least one prior systemic therapy | adagrasib | 12/12/2022 | Single Enantiomer | 2 | C |
| **Rezlidhia** | To treat adults with relapsed or refractory acute myeloid leukemia with a susceptible isocitrate dehydrogenase-1 (IDH1) mutation | olutasidenib | 01/12/2022 | Single Enantiomer | 1 | C |
| **Tzield** | To delay the onset of stage 3 type 1 diabetesPress Release | teplizumab-mzwv | 18/11/2022 | Biologic | - | - |

| | | | | | | |
|---|---|---|---|---|---|---|
| **Elahere** | To treat patients with recurrent ovarian cancer that is resistant to platinum therapy | mirvetuximab soravtansine-gynx | 14/11/2022 | Biologic | - | - |
| **Tecvayli** | To treat relapsed or refractory multiple myeloma among adults who have received at least four specific lines of therapy | teclistamab-cqyv | 25/10/2022 | Biologic | - | - |
| **Imjudo** | To treat unresectable hepatocellular carcinoma | tremelimumab | 21/10/2022 | Biologic | - | - |
| **Lytgobi** | To treat intrahepatic cholangiocarcinoma harboring fibroblast growth factor receptor 2 (FGFR2) gene fusions or other rearrangements | futibatinib | 30/09/2022 | Single Enantiomer | 1 | C |
| **Omlonti** | To reduce elevated intraocular pressure in patients with open-angle glaucoma or ocular hypertension | oomidenepag isopropyl ophthalmic solution | 22/09/2022 | Achiral | - | - |
| **Elucirem** | To detect and visualize lesions, together with MRI, with abnormal vascularity in the central nervous system and the body | gadopiclenol | 21/09/2022 | Racemate | 6 | C |
| **Terlivaz** | To improve kidney function in adults with hepatorenal syndrome with rapid reduction in kidney function | terlipressin | 14/09/2022 | Biologic | - | - |
| **Rolvedon** | To decrease the incidence of infection in patients with non-myeloid malignancies receiving myelosuppressive anti-cancer drugs associated with clinically significant incidence of febrile neutropenia | eflapegrastim | 09/09/2022 | Biologic | - | - |
| **Sotyktu** | To treat moderate-to-severe plaque psoriasis | deucravacitinib | 09/09/2022 | Achiral | - | - |
| **Daxxify** | To treat moderate-to-severe glabellar lines associated with corrugator and/or procerus muscle activity | daxibotulinumtoixnA-lanm | 07/09/2022 | Biologic | - | - |
| **Spevigo** | To treat generalized pustular psoriasis flares | spesolimab-sbzo | 01/09/2022 | Biologic | - | - |
| **Xenpozyme** | To treat Acid Sphingomyelinase DeficiencyPress Release | Olipudase alfa | 31/08/2022 | Biologic | - | - |
| **Amvuttra** | To treat polyneuropathy of hereditary transthyretin-mediated amyloidosis | vutrisiran | 13/06/2022 | Biologic | - | - |
| **Vtama** | To treat plaque psoriasis | tapinarof | 23/05/2022 | Achiral | - | - |
| **Mounjaro** | To improve blood sugar control in diabetes, in addition to diet and exercise Press Release | tirzepatide | 13/05/2022 | Biologic | - | - |
| **Voquezna** | To treat Helicobacter pylori infection | vonoprazan, amoxicillin, and clarithromycin | 03/05/2022 | Achiral | - | - |

| | | | | | | |
|---|---|---|---|---|---|---|
| **Camzyos** | To treat certain classes of obstructive hypertrophic cardiomyopathy | mavacamten | 28/04/2022 | Single Enantiomer | 1 | C |
| **Vivjoa** | To reduce the incidence of recurrent vulvovaginal candidiasis (RVVC) in females with a history of RVVC who are not of reproductive potential | oteseconazole | 26/04/2022 | Single Enantiomer | 1 | C |
| **Pluvicto** | To treat prostate-specific membrane antigen-positive metastatic castration-resistant prostate cancer following other therapies | lutetium (177Lu) vipivotide tetraxetan | 23/03/2022 | Biologic | - | - |
| **Opdualag** | To treat unresectable or metastatic melanoma | nivolumab and relatlimab-rmbw | 18/03/2022 | Biologic | - | - |
| **Ztalmy** | To treat seizures in cyclin-dependent kinase-like 5 deficiency disorder | ganaxolone | 18/03/2022 | Single Enantiomer | 8 | C |
| **Vonjo** | To treat intermediate or high-risk primary or secondary myelofibrosis in adults with low platelets | pacritinib | 28/02/2022 | Achiral | - | - |
| **Pyrukynd** | To treat hemolytic anemia in pyruvate kinase deficiency | mitapivat | 17/02/2022 | Achiral | - | - |
| **Enjaymo** | To decrease the need for red blood cell transfusion due to hemolysis in cold agglutinin disease | sutimlimab-jome | 04/02/2022 | Biologic | - | - |
| **Vabysmo** | To treat neovascular (wet) aged-related macular degeneration and diabetic macular edema | faricimab-svoa | 28/01/2022 | Biologic | - | - |
| **Kimmtrak** | To treat unresectable or metastatic uveal melanoma | tebentafusp-tebn | 25/01/2022 | Biologic | - | - |
| **Cibinqo** | To treat refractory, moderate-to-severe atopic dermatitis | abrocitinib | 14/01/2022 | Achiral | - | - |
| **Quviviq** | To treat insomnia | daridorexant | 07/01/2022 | Single Enantiomer | 1 | C |
| **Adbry** | To treat moderate-to-severe atopic dermatitis | tralokinumab-ldrm | 27/12/..021 | Biologic | - | - |
| **Leqvio** | To treat heterozygous familial hypercholesterolemia or clinical atherosclerotic cardiovascular disease as an add-on therapy | inclisiran | 22/12/2021 | Biologic | - | - |
| **Vyvgart** | To treat generalized myasthenia gravis | efgartigimod alfa-fcab | 17/12/2021 | Biologic | - | - |
| **Tezspire** | To treat severe asthma as an add-on maintenance therapy | tezepelumab-ekko | 17/12/2021 | Biologic | - | - |
| **Cytalux** | To help identify ovarian cancer lesions | pafolacianine | 29/11/2021 | Single Enantiomer | 1 | C |
| **Livtencity** | To treat post-transplant cytomegalovirus (CMV) infection/disease that does not respond (with or without | maribavir | 23/11/2021 | Single Enantiomer | 4 | C |

| | | | | | | |
|---|---|---|---|---|---|---|
| | genetic mutations that cause resistance) to available antiviral treatment for CMV | | | | | |
| **Voxzogo** | To improve growth in children five years of age and older with achondroplasia and open epiphyses | vosoritide | 19/11/2021 | Biologic | - | - |
| **Besremi** | To treat polycythemia vera, a blood disease that causes the overproduction of red blood cells | ropeginterferon alfa-2b-njft | 12/11/2021 | Biologic | - | - |
| **Scemblix** | To treat Philadelphia chromosome-positive chronic myeloid leukemia with disease that meets certain criteria | asciminib | 29/10/2021 | Single Enantiomer | 1 | C |
| **Tavneos** | To treat severe active anti-neutrophil cytoplasmic autoantibody-associated vasculitis (granulomatosis with polyangiitis and microscopic polyangiitis) in combination with standard therapy, including glucocorticoids | avacopan | 07/10/2021 | Single Enantiomer | 2 | C |
| **Livmarli** | To treat cholestatic pruritus associated with Alagille syndrome | maralixibat | 29/09/2021 | Single Enantiomer | 2 | C |
| **Qulipta** | To prevent episodic migraines | atogepant | 28/09/2021 | Single Enantiomer | 4 | C |
| **Tivdak** | To treat recurrent or metastatic cervical cancer with disease progression on or after chemotherapy | tisotumab vedotin-tftv | 20/09/2021 | Biologic | - | - |
| **Exkivity** | To treat locally advanced or metastatic non-small cell lung cancer with epidermal growth factor receptor exon 20 insertion mutations | mobocertinib | 15/09/2021 | Achiral | - | - |
| **Skytrofa** | To treat short stature due to inadequate secretion of endogenous growth hormone | lonapegsomatropin-tcgd | 25/08/2021 | Biologic | - | - |
| **Korsuva** | To treat moderate-to-severe pruritus associated with chronic kidney disease in certain populations | difelikefalin | 23/08/2021 | Biologic | - | - |
| **Welireg** | To treat von Hippel-Lindau disease under certain conditions | belzutifan | 13/08/2021 | Single Enantiomer | 3 | C |
| **Nexviazyme** | To treat late-onset Pompe disease | avalglucosidase alfa-ngpt | 06/08/2021 | Biologic | - | - |
| **Saphnelo** | To treat moderate-to severe systemic lupus erythematousus along with standard therapy | anifrolumab-fnia | 30/07/2021 | Biologic | - | - |
| **Bylvay** | To treat pruritus | odevixibat | 20/07/2021 | Single Enantiomer | 2 | C |
| **Rezurock** | To treat chronic graft-versus-host disease after failure of at least two prior lines of systemic therapy | belumosudil | 16/07/2021 | Achiral | - | - |

| | | | | | | |
|---|---|---|---|---|---|---|
| **fexinidazole** | To treat human African trypanosomiasis caused by the parasite Trypanosoma brucei gambiense | fexinidazole | 16/07/2021 | Achiral | - | - |
| **Kerendia** | To reduce the risk of kidney and heart complications in chronic kidney disease associated with type 2 diabetes | finerenone | 09/07/2021 | Single Enantiomer | 1 | C |
| **Rylaze** | To treat acute lymphoblastic leukemia and lymphoblastic lymphoma in patients who are allergic to E. coli-derived asparaginase products, as a component of a chemotherapy regimen | asparaginase erwinia chrysanthemi (recombinant)-rywn | 30/06/2021 | Biologic | - | - |
| **Aduhelm** | To treat Alzheimer's disease | aducanumab-avwa | 07/06/2021 | Biologic | - | - |
| **Brexafemme** | To treat vulvovaginal candidiasis | ibrexafungerp | 01/06/2021 | Single Enantiomer | 12 | C |
| **Lybalvi** | To treat schizophrenia and certain aspects of bipolar I disorder | olanzapine and samidorphan | 28/05/2021 | Single Enantiomer | 3 | C |
| **Truseltiq** | To treat cholangiocarcinoma whose disease meets certain criteria | infigratinib | 28/05/2021 | Achiral | - | - |
| **Lumakras** | To treat types of non-small cell lung cancer | sotorasib | 28/05/2021 | Single Enantiomer | 1 | C |
| **Pylarify** | To identify prostate-specific membrane antigen-positive lesions in prostate cancer | piflufolastat F 18 | 26/05/2021 | Single Enantiomer | 2 | C |
| **Rybrevant** | To treat a subset of non-small cell lung cancer | amivantamab-vmjw | 21/05/2021 | Biologic | - | - |
| **Empaveli** | To treat paroxysmal nocturnal hemoglobinuria | pegcetacoplan | 5/14/2021 | Biologic | - | - |
| **Zynlonta** | To treat certain types of relapsed or refractory large B-cell lymphoma | loncastuximab tesirine-lpyl | 23/04/2021 | Biologic | - | - |
| **Jemperli** | To treat endometrial cancer | dostarlimab-gxly | 23/04/2021 | Biologic | - | - |
| **Nextstellis** | To prevent pregnancy | drospirenone and **estetrol** | 15/04/2021 | Single Enantiomer | 7 | C |
| **Qelbree** | To treat attention deficit hyperactivity disorder | viloxazine | 02/04/2021 | Racemate | 1 | C |
| **Zegalogue** | To treat severe hypoglycemia | dasiglucagon | 22/03/2021 | Biologic | - | - |
| **Ponvory** | To treat relapsing forms of multiple sclerosis | ponesimod | 18/03/2021 | Single Enantiomer | 1 | C |
| **Fotivda** | To treat renal cell carcinoma | tivozanib | 10/03/2021 | Achiral | - | - |
| **Azstarys** | To treat attention deficit hyperactivity disorder | **serdexmethylphenidate** and dexmethylphenidate | 02/03/2021 | Single Enantiomer | 3 | C |

| | | | | | | |
|---|---|---|---|---|---|---|
| **Pepaxto** | To treat relapsed or refractory multiple myeloma | melphalan flufenamide | 26/02/2021 | Single Enantiomer | 2 | C |
| **Nulibry** | To reduce the risk of mortality in molybdenum cofactor deficiency Type A | fosdenopterin | 26/02/2021 | Single Enantiomer | 4 | C |
| **Amondys 45** | To treat Duchenne muscular dystrophy | casimersen | 25/02/2021 | Biologic | - | - |
| **Cosela** | To mitigate chemotherapy-induced myelosuppression in small cell lung cancer | trilacicilib | 12/02/2021 | Achiral | - | - |
| **Evkeeza** | To treat homozygous familial hypercholesterolemia | evinacumab-dgnb | 11/02/2021 | Biologic | - | - |
| **Ukoniq** | To treat marginal zone lymphoma and follicular lymphoma | umbralisib | 05/02/2021 | Single Enantiomer | 1 | C |
| **Tepmetko** | To treat non-small cell lung cancer | tepotinib | 03/02/2021 | Achiral | - | - |
| **Lupkynis** | To treat lupus nephritis | voclosporin | 22/01/2021 | Single Enantiomer | 12 | C |
| **Cabenuva** | To treat HIV | **cabotegravir** and rilpivirine (co-packaged) | 21/01/2021 | Single Enantiomer | 2 | C |
| **Verquvo** | To mitigate the risk of cardiovascular death and hospitalization for chronic heart failure | vericiguat | 19/01/2021 | Achiral | - | - |
| **Gemtesa** | To treat overactive bladder | vibegron | 23/12/2020 | Single Enantiomer | 4 | C |
| **Ebanga** | To treat ebola | ansuvimab-zykl | 21/12/2020 | Biologic | - | - |
| **Orgovyx** | To treat advanced prostate cancer | relugolix | 18/12/2020 | Achiral | - | - |
| **Margenza** | To treat HER2+ breast cancer | margetuximab (anti-HER2 mAb | 16/12/2020 | Biologic | - | - |
| **Klisyri** | To treat actinic Keratosis of the face or scalp | tirbanibulin | 14/12/2020 | Achiral | - | - |
| **Cerianna** | To treat patients with hereditary angioedema | berotralstat | 03/12/2020 | Single Enantiomer | 1 | C |
| **Gallium 68 PSMA-11** | For detection and localization of prostate cancer | Gallium 68 PSMA-11 | 01/12/2020 | Single Enantiomer | 2 | C |
| **Danyelza** | To treat high-risk refractory or relapsed neuroblastoma | naxitamab-gqgk | 25/11/2020 | Biologic | - | - |
| **Imcivree** | To treat obesity and the control of hunger associated with pro-opiomelanocortin deficiency, a rare disorder that causes severe obesity that begins at an early age | setmelanotide | 25/11/2020 | Biologic | - | - |
| **Oxlumo** | To treat hyperoxaluria type 1 | lumasiran | 23/11/2020 | Biologic | - | - |

| | | | | | | |
|---|---|---|---|---|---|---|
| **Zokinvy** | To treat rare conditions related to premature aging | lonafarnib | 20/11/2020 | Single Enantiomer | 1 | C |
| **Veklury** | To treat COVID-19 | remdesivir | 22/10/2020 | Single Enantiomer | 6 | C |
| **Inmazeb** | To treat ebola virus | atoltivimab, maftivimab, and odesivimab-ebgn | 14/10/2020 | Biologic | - | - |
| **Gavreto** | To treat non-small lung cancer | pralsetinib | 04/09/2020 | Single Enantiomer | 1 | C |
| **Detectnet** | To help detect certain types of neuroendocrine tumors | copper Cu 64 dotatate injection | 03/09/2020 | Single Enantiomer | 10 | C |
| **Sogroya** | Growth hormone | somapacitan-beco | 28/08/2020 | Biologic | - | - |
| **Winlevi** | To treat acne | clascoterone | 26/08/2020 | Single Enantiomer | 6 | C |
| **Enspryng** | To treat neuromyelitis optica spectrum disorder | satralizumab-mwge | 14/08/2020 | Biologic | - | - |
| **Viltepso** | To treat Duchenne muscular dystrophy | viltolarsen | 12/08/2020 | Biologic | - | - |
| **Olinvyk** | To manage acute pain in certain adults | oliceridine | 07/08/2020 | Single Enantiomer | 1 | C |
| **Evrysdi** | To treat spinal muscular atrophy | risdiplam | 07/08/2020 | Achiral | - | - |
| **Lampit** | To treat Chagas disease in certain pediatric patients younger than age 18 | nifurtimox | 06/08/2020 | Racemate | 1 | C |
| **Blenrep** | To treat multiple myeloma | belantamab mafodotin-blmf | 05/08/2020 | Biologic | - | - |
| **Monjuvi** | To treat relapsed or refractory diffuse large B-cell lymphoma | tafasitamab-cxix | 31/07/2020 | Biologic | - | - |
| **Xeglyze** | To treat head lice | abametapir | 24/07/2020 | Achiral | - | - |
| **Inqovi** | To treat adult patients with myelodysplastic syndromes | decitabine and **cedazuridine** | 07/07/2020 | Single Enantiomer | 4 | C |
| **Rukobia** | To treat HIV | fostemsavir | 02/07/2020 | Achiral | - | - |
| **Byfavo** | For sedation | remimazolam | 02/07/2020 | Single Enantiomer | 1 | C |
| **Dojolvi** | To treat molecularly long-chain fatty acid oxidation disorders | triheptanoin | 30/06/2020 | Achiral | - | - |
| **Zepzelca** | To treat metastatic small cell lung cancer | lurbinectedin | 15/06/2020 | Single Enantiomer | 7 | C |
| **Uplizna** | To treat neuromyelitis optica spectrum disorder | inebilizumab-cdon | 11/06/2020 | Biologic | - | - |
| **Tauvid** | Diagnostic agent for patients with Alzheimer’s disease | flortaucipir F18 | 28/05/2020 | Achiral | - | - |

| | | | | | | |
|---|---|---|---|---|---|---|
| **Artesunate** | To treat severe malaria | artesunate | 26/05/2020 | Single Enantiomer | 8 | C |
| **Cerianna** | Diagnostic imaging agent for certain patients with breast cancer | fluoroestradiol F18 | 20/05/2020 | Single Enantiomer | 6 | C |
| **Qinlock** | To treat advanced gastrointestinal-stromal tumors | ripretinib | 15/05/2020 | Achiral | - | - |
| **Retevmo** | To treat lung and thyroid cancers | selpercatinib | 08/05/2020 | Achiral | - | - |
| **Tabrecta** | To treat patients with non small cell lung cancer | capmatinib | 06/05/2020 | Achiral | - | - |
| **Ongentys** | To treat patients with Parkinson’s disease experiencing “off” episodes | opicapone | 24/04/2020 | Achiral | - | - |
| **Trodelvy** | To treat adult patients with metastatic triple-negative breast cancer who received at least two prior therapies for metastatic disease | sacituzumab govitecan-hziy | 22/04/2020 | Biologic | - | - |
| **Pemazyre** | To treat certain patients with cholangiocarcinoma, a rare form of cancer that forms in bile ducts | pemigatinib | 17/04/2020 | Achiral | - | - |
| **Tukysa** | To treat advanced unresectable or metastatic HER2-positive breast cancer | tucatinib | 17/04/2020 | Achiral | - | - |
| **Koselugo** | To treat neurofibromatosis type 1, a genetic disorder of the nervous system causing tumors to grow on nerves | selumetinib | 10/04/2020 | Achiral | - | - |
| **Zeposia** | To treat relapsing forms of multiple sclerosis | ozanimod | 25/03/2020 | Single Enantiomer | 1 | C |
| **Isturisa** | To treat adults with Cushing’s disease who either cannot undergo pituitary gland surgery or have undergone the surgery but still have the disease | osilodrostat | 06/03/2020 | Single Enantiomer | 1 | C |
| **Sarclisa** | To treat multiple myloma | isatuximab | 02/03/2020 | Biologic | - | - |
| **Nurtec ODT** | To treat migraine | rimegepant | 27/02/2020 | Single Enantiomer | 3 | C |
| **Barhemsys** | To help prevent nausea and vomiting after surgery | amisulpride | 26/02/2020 | Racemate | 1 | C |
| **Vyepti** | For the preventive treatment of migraine in adults | eptinezumab-jjmr | 21/02/2020 | Biologic | - | - |
| **Nexletol** | To treat adults with heterozygous familial hypercholesterolemia or established atherosclerotic cardiovascular disease who require additional lowering of LDL-C | bempedoic acid | 21/02/2020 | Achiral | - | - |

| | | | | | | |
|---|---|---|---|---|---|---|
| **Pizensy** | To treat chronic idiopathic constipation (CIC) in adults | lactitol | 12/02/2020 | Single Enantiomer | 6 | C |
| **Tazverik** | To treat epithelioid sarcoma | tazemetostat | 23/01/2020 | Achiral | - | - |
| **Tepezza** | To treat Thyroid eye disease | teprotumumab-trbw | 21/01/2020 | Biologic | - | - |
| **Ayvakit** | To treat adults with unresectable or metastatic gastrointestinal stromal tumor (GIST) | avapritinib | 09/01/2020 | Single Enantiomer | 1 | C |

# EMA NAS Classification (2013-2022)

*Table S10. All medicines containing new active substances (NASs) approved for marketing authorisation by the EMA from 2013-2022 classified as either biologic, achiral, single enantiomer, racemate, polymer or herbal substance.*

*Where more than one active substance is listed, the NAS(s) is shown in bold. Where a medicine contains more than one NAS, an additional entry has been added for each additional NAS.*

*Note: an Excel file containing these tables is also available as Supporting Information.*

| Medicine Name | Specific Therapeutic Area | Active Substance | Marketing Authorisation Date | Drug Type/ Chirality | No. Stereo-centres | Type of Stereo-centres |
|---|---|---|---|---|---|---|
| **Krystexxa** | Gout | pegloticase | 8/01/2013 | Biologic | - | - |
| **Bexsero** | Meningitis, Meningococcal | outer membrane vesicles from neisseria meningitidis group b (strain nz 98/254), recombinant neisseria meningitidis group b fhbp fusion protein, recombinant neisseria meningitidis group b nada protein, recombinant neisseria meningitidis group b nhba fusion protein | 13/01/2013 | Biologic | - | - |
| **Amyvid** | Radionuclide Imaging | florbetapir (18f) | 14/01/2013 | Achiral | - | - |
| **Tresiba** | Diabetes Mellitus | insulin degludec | 20/01/2013 | Biologic | - | - |
| **BindRen** | Hyperphosphatemia | colestilan | 21/01/2013 | Achiral | - | - |
| **Ryzodeg** | Diabetes Mellitus | **insulin aspart**, insulin degludec | 21/01/2013 | Biologic | - | - |
| **Lyxumia** | Diabetes Mellitus, Type 2 | lixisenatide | 31/01/2013 | Biologic | - | - |
| **Zaltrap** | Colorectal Neoplasms | aflibercept | 1/02/2013 | Biologic | - | - |
| **Selincro** | Alcohol-Related Disorders | nalmefene hydrochloride dihydrate | 24/02/2013 | Single Enantiomer | 4 | C |

| | | | | | | |
|---|---|---|---|---|---|---|
| **Perjeta** | Breast Neoplasms | pertuzumab | 4/03/2013 | Biologic | - | - |
| **Jetrea** | Retinal Diseases | ocriplasmin | 13/03/2013 | Biologic | - | - |
| **Bosulif** | Leukemia, Myeloid | bosutinib (as monohydrate) | 27/03/2013 | Achiral | - | - |
| **Stribild** | HIV Infections | **elvitegravir, cobicistat**, emtricitabine, tenofovir disoproxil fumarate | 24/05/2013 | X | - | - |
| **Iclusig** | Leukemia, Myeloid; Leukemia, Lymphoid | ponatinib | 1/07/2013 | Achiral | - | - |
| **Lonquex** | Neutropenia | lipegfilgrastim | 25/07/2013 | Biologic | - | - |
| **Lojuxta** | Hypercholesterolemia | lomitapide | 31/07/2013 | Achiral | - | - |
| **Imvanex** | Smallpox Vaccine; Monkeypox virus | modified vaccinia ankara - bavarian nordic (mva-bn) virus | 31/07/2013 | Biologic | - | - |
| **Imnovid** | Multiple Myeloma | pomalidomide | 5/08/2013 | Racemate | 1 | C |
| **Tafinlar** | Melanoma | dabrafenib mesilate | 26/08/2013 | Achiral | - | - |
| **Stivarga** | Colorectal Neoplasms | regorafenib | 26/08/2013 | Achiral | - | - |
| **Aubagio** | Multiple Sclerosis | teriflunomide | 26/08/2013 | Achiral | - | - |
| **Provenge** | Prostatic Neoplasms | autologous peripheral-blood mononuclear cells including a minimum of 50 million autologous cd54+ cells activated with prostatic acid phosphatase granulocyte-macrophage colony-stimulating factor | 6/09/2013 | Biologic | - | - |
| **Vipidia** | Diabetes Mellitus, Type 2 | alogliptin | 18/09/2013 | Single Enantiomer | 1 | C |
| **Vipdomet** | Diabetes Mellitus, Type 2 | **alogliptin benzoate**, metformin hydrochloride | 18/09/2013 | Single Enantiomer | 1 | C |
| **Incresync** | Diabetes Mellitus, Type 2 | alogliptin, pioglitazone | 19/09/2013 | Single Enantiomer | 1 | C |
| **Tybost** | HIV Infections | cobicistat | 19/09/2013 | Single Enantiomer | 3 | C |
| **Giotrif** | Carcinoma, Non-Small-Cell Lung | afatinib | 25/09/2013 | Single Enantiomer | 1 | C |

| | | | | | | |
|---|---|---|---|---|---|---|
| **Defitelio** | Hepatic Veno-Occlusive Disease | defibrotide | 18/10/2013 | Biologic | - | - |
| **Vitekta** | HIV Infections | elvitegravir | 13/11/2013 | Single Enantiomer | 1 | C |
| **Xofigo** | Prostatic Neoplasms | radium (223ra) dichloride | 13/11/2013 | Achiral | - | - |
| **NovoEight** | Hemophilia A | turoctocog alfa | 13/11/2013 | Biologic | - | - |
| **Invokana** | Diabetes Mellitus, Type 2 | canagliflozin | 15/11/2013 | Single Enantiomer | 5 | C |
| **Kadcyla** | Breast Neoplasms | trastuzumab emtansine | 15/11/2013 | Biologic | - | - |
| **Fluenz Tetra** | Influenza, Human | a/victoria/2570/2019 (h1n1)pdm09 - like strain (a/victoria/1/2020, medi 340505) a/darwin/9/2021 (h3n2) - like strain (a/norway/16606/2021, medi 355293) b/austria/1359417/2021 - like strain (b/austria/1359417/2021, medi 355292) b/phuket/3073/2013 - like strain (b/phuket/3073/2013, medi 306444 | 4/12/2013 | Biologic | - | - |
| **Brintellix** | Depressive Disorder, Major | vortioxetine | 18/12/2013 | Achiral | - | - |
| **Opsumit** | Hypertension, Pulmonary | macitentan | 20/12/2013 | Achiral | - | - |
| **Tivicay** | HIV Infections | dolutegravir | 16/01/2014 | Single Enantiomer | 2 | C |
| **Sovaldi** | Hepatitis C, Chronic | sofosbuvir | 16/01/2014 | Single Enantiomer | 6 | 5C, 1P |
| **Tecfidera** | Multiple Sclerosis | dimethyl fumarate | 30/01/2014 | Achiral | - | - |
| **Neuraceq** | Radionuclide Imaging; Alzheimer Disease | florbetaben (18f) | 20/02/2014 | Achiral | - | - |
| **Sirturo** | Tuberculosis, Multidrug-Resistant | bedaquiline fumarate | 5/03/2014 | Single Enantiomer | 2 | C |
| **Eperzan** | Diabetes Mellitus, Type 2 | albiglutide | 20/03/2014 | Biologic | - | - |
| **Cometriq** | Thyroid Neoplasms | cabozantinib | 21/03/2014 | Achiral | - | - |
| **Latuda** | Schizophrenia | lurasidone | 21/03/2014 | Single Enantiomer | 6 | C |

| | | | | | | |
|---|---|---|---|---|---|---|
| **Adempas** | Hypertension, Pulmonary | riociguat | 27/03/2014 | Achiral | - | - |
| **Vokanamet** | Diabetes Mellitus, Type 2 | **canagliflozin**, metformin hydrochloride | 23/04/2014 | Single Enantiomer | 5 | C |
| **Vimizim** | Mucopolysaccharidosis IV | recombinant human n-acetylgalactosamine-6-sulfatase | 27/04/2014 | Biologic | - | - |
| **Incruse Ellipta** | Pulmonary Disease, Chronic Obstructive | umeclidinium bromide | 28/04/2014 | Achiral | - | - |
| **Anoro Ellipta** | Pulmonary Disease, Chronic Obstructive | **umeclidinium bromide,** vilanterol trifenatate | 8/05/2014 | Achiral | - | - |
| **Anoro Ellipta** | Pulmonary Disease, Chronic Obstructive | umeclidinium bromide, **vilanterol trifenatate** | 8/05/2014 | Single Enantiomer | 1 | C |
| **Laventair Ellipta** | Pulmonary Disease, Chronic Obstructive | umeclidinium bromide, vilanterol | 8/05/2014 | | - | - |
| **Olysio** | Hepatitis C, Chronic | simeprevir | 14/05/2014 | Single Enantiomer | 5 | C |
| **Entyvio** | Colitis, Ulcerative; Crohn Disease | vedolizumab | 22/05/2014 | Biologic | - | - |
| **Sylvant** | Giant Lymph Node Hyperplasia | siltuximab | 22/05/2014 | Biologic | - | - |
| **Jardiance** | Diabetes Mellitus, Type 2 | empagliflozin | 22/05/2014 | Single Enantiomer | 6 | C |
| **Mekinist** | Melanoma | trametinib | 30/06/2014 | Achiral | - | - |
| **Plegridy** | Multiple Sclerosis | peginterferon beta-1a | 18/07/2014 | Biologic | - | - |
| **Nuwiq** | Hemophilia A | simoctocog alfa | 22/07/2014 | Biologic | - | - |
| **Gazyvaro** | Leukemia, Lymphocytic, Chronic, B-Cell | obinutuzumab | 22/07/2014 | Biologic | - | - |
| **Translarna** | Muscular Dystrophy, Duchenne | ataluren | 31/07/2014 | Achiral | - | - |
| **Vizamyl** | Radionuclide Imaging; Alzheimer Disease | flutemetamol (18f) | 22/08/2014 | Achiral | - | - |
| **Daklinza** | Hepatitis C, Chronic | daclatasvir dihydrochloride | 22/08/2014 | Single Enantiomer | 4 | C |

| | | | | | | |
|---|---|---|---|---|---|---|
| **Velphoro** | Hyperphosphatemia; Renal Dialysis | sucroferric oxyhydroxide | 26/08/2014 | Biologic | - | - |
| **Triumeq** | HIV Infections | **dolutegravir sodium**, lamivudine, abacavir (as sulfate) | 31/08/2014 | Single Enantiomer | 2 | C |
| **Zydelig** | Lymphoma, Non-Hodgkin; Leukemia, Lymphocytic, Chronic, B-Cell | idelalisib | 18/09/2014 | Single Enantiomer | 1 | C |
| **Imbruvica** | Lymphoma, Mantle-Cell; Leukemia, Lymphocytic, Chronic, B-Cell | ibrutinib | 21/10/2014 | Single Enantiomer | 1 | C |
| **Harvoni** | Hepatitis C, Chronic | **ledipasvir**, sofosbuvir | 17/11/2014 | Single Enantiomer | 6 | C |
| **Lymphoseek** | Radionuclide Imaging | tilmanocept | 19/11/2014 | Polymer | - | - |
| **Trulicity** | Diabetes Mellitus, Type 2 | dulaglutide | 21/11/2014 | Biologic | - | - |
| **Vargatef** | Carcinoma, Non-Small-Cell Lung | nintedanib | 21/11/2014 | Achiral | - | - |
| **Moventig** | Constipation; Opioid-Related Disorders | naloxegol oxalate | 7/12/2014 | Single Enantiomer | 5 | C |
| **Lynparza** | Ovarian Neoplasms | olaparib | 16/12/2014 | Achiral | - | - |
| **Cyramza** | Stomach Neoplasms | ramucirumab | 19/12/2014 | Biologic | - | - |
| **Scenesse** | Protoporphyria, Erythropoietic | afamelanotide | 22/12/2014 | Biologic | - | - |
| **Tenkasi** | Soft Tissue Infections; Skin Diseases, Bacterial | oritavancin diphosphate | 18/03/2015 | Biologic | - | - |
| **Kengrexal** | Acute Coronary Syndrome; Vascular Surgical Procedures | cangrelor | 23/03/2015 | Single Enantiomer | 4 | C |
| **Sivextro** | Soft Tissue Infections; Skin Diseases, Bacterial | tedizolid phosphate | 23/03/2015 | Single Enantiomer | 1 | C |
| **Zykadia** | Carcinoma, Non-Small-Cell Lung | ceritinib | 6/05/2015 | Achiral | - | - |
| **Lenvima** | Thyroid Neoplasms | lenvatinib mesilate | 28/05/2015 | Achiral | - | - |

| | | | | | | |
|---|---|---|---|---|---|---|
| **Gardasil 9** | Condylomata Acuminata; Papillomavirus Infections; Immunization; Uterine Cervical Dysplasia | human papillomavirus vaccine [types 6, 11, 16, 18, 31, 33, 45, 52, 58] (recombinant, adsorbed) | 10/06/2015 | Biologic | - | - |
| **Opdivo** | Melanoma; Hodgkin Disease; Carcinoma, Renal Cell; Carcinoma, Non-Small-Cell Lung; Carcinoma, Transitional Cell; Squamous Cell Carcinoma of Head and Neck; Urologic Neoplasms; Mesothelioma; Colorectal Neoplasms | nivolumab | 19/06/2015 | Biologic | - | - |
| **Lixiana** | Stroke; Venous Thromboembolism | edoxaban tosilate | 19/06/2015 | Single Enantiomer | 3 | C |
| **Hetlioz** | Sleep Disorders, Circadian Rhythm | tasimelteon | 3/07/2015 | Single Enantiomer | 2 | C |
| **Keytruda** | Melanoma; Hodgkin Disease; Carcinoma, Renal Cell; Carcinoma, Non-Small-Cell Lung; Carcinoma, Transitional Cell; Squamous Cell Carcinoma of Head and Neck; Urologic Neoplasms; Endometrial Neoplasms | pembrolizumab | 17/07/2015 | Biologic | - | - |
| **Repatha** | Dyslipidemias; Hypercholesterolemia | evolocumab | 17/07/2015 | Biologic | - | - |
| **Nivolumab BMS** | Carcinoma, Non-Small-Cell Lung | nivolumab | 20/07/2015 | Biologic | - | - |
| **Odomzo** | Carcinoma, Basal Cell | sonidegib diphosphate | 14/08/2015 | Achiral | - | - |
| **Unituxin** | Neuroblastoma | dinutuximab | 14/08/2015 | Biologic | - | - |

| | | | | | | |
|---|---|---|---|---|---|---|
| **Farydak** | Multiple Myeloma | panobinostat lactate anhydrous | 28/08/2015 | Racemate | 1/1 | C/C |
| **Kanuma** | Lipid Metabolism, Inborn Errors | sebelipase alfa | 28/08/2015 | Biologic | - | - |
| **Strensiq** | Hypophosphatasia | asfotase alfa | 28/08/2015 | Biologic | - | - |
| **Zerbaxa** | Bacterial Infections | **ceftolozane sulfate**, tazobactam sodium | 18/09/2015 | Single Enantiomer | 2 | C |
| **Praluent** | Dyslipidemias | alirocumab | 23/09/2015 | Biologic | - | - |
| **Fexeric** | Hyperphosphatemia; Renal Dialysis | ferric citrate coordination complex | 23/09/2015 | Achiral | - | - |
| **Cresemba** | Aspergillosis | isavuconazole | 15/10/2015 | Epimers | 3 | C |
| **Obizur** | Hemophilia A | susoctocog alfa | 11/11/2015 | Biologic | - | - |
| **Elocta** | Hemophilia A | efmoroctocog alfa | 18/11/2015 | Biologic | - | - |
| **Orkambi** | Cystic Fibrosis | **lumacaftor**, ivacaftor | 18/11/2015 | Achiral | - | - |
| **Kyprolis** | Multiple Myeloma | carfilzomib | 19/11/2015 | Single Enantiomer | 5 | C |
| **Entresto** | Heart Failure | sacubitril, valsartan | 19/11/2015 | Single Enantiomer | 3 | C |
| **Genvoya** | HIV Infections | elvitegravir, cobicistat, emtricitabine, **tenofovir alafenamide** | 19/11/2015 | Single Enantiomer | 3 | 1P, 2C |
| **Cotellic** | Melanoma | cobimetinib hemifumarate | 20/11/2015 | Single Enantiomer | 1 | C |
| **Praxbind** | Hemorrhage | idarucizumab | 20/11/2015 | Biologic | - | - |
| **Blincyto** | Precursor Cell Lymphoblastic Leukemia-Lymphoma | blinatumomab | 23/11/2015 | Biologic | - | - |
| **Ravicti** | Urea Cycle Disorders, Inborn | glycerol phenylbutyrate | 26/11/2015 | Achiral | - | - |
| **Nucala** | Asthma | mepolizumab | 1/12/2015 | Biologic | - | - |
| **Imlygic** | Melanoma | talimogene laherparepvec | 16/12/2015 | Biologic | - | - |
| **Briviact** | Epilepsy | brivaracetam | 13/01/2016 | Single Enantiomer | 2 | C |

| | | | | | | |
|---|---|---|---|---|---|---|
| **Episalvan** | Wounds and Injuries; Wound Healing | betulae cortex | 14/01/2016 | Herbal Substance | - | - |
| **Tagrisso** | Carcinoma, Non-Small-Cell Lung | osimertinib mesilate | 1/02/2016 | Achiral | - | - |
| **Portrazza** | Carcinoma, Non-Small-Cell Lung | necitumumab | 15/02/2016 | Biologic | - | - |
| **Zurampic** | Hyperuricemia | lesinurad | 18/02/2016 | Racemate | 0 | n/a |
| **Wakix** | Narcolepsy | pitolisant | 31/03/2016 | Achiral | - | - |
| **Lonsurf** | Colorectal Neoplasms | trifluridine, **tipiracil hydrochloride** | 25/04/2016 | Achiral | - | - |
| **Taltz** | Psoriasis | ixekizumab | 25/04/2016 | Biologic | - | - |
| **Empliciti** | Multiple Myeloma | elotuzumab | 11/05/2016 | Biologic | - | - |
| **Idelvion** | Hemophilia B | albutrepenonacog alfa | 11/05/2016 | Biologic | - | - |
| **Uptravi** | Hypertension, Pulmonary | selexipag | 12/05/2016 | Achiral | - | - |
| **Alprolix** | Hemophilia B | eftrenonacog alfa | 12/05/2016 | Biologic | - | - |
| **Darzalex** | Multiple Myeloma | daratumumab | 20/05/2016 | Biologic | - | - |
| **Pandemic influenza vaccine (H5N1) MedImmune** | Influenza, Human | reassortant influenza virus (live attenuated) of the following strain: a/vietnam/1203/2004 (h5n1) strain | 20/05/2016 | Biologic | - | - |
| **Galafold** | Fabry Disease | migalastat hydrochloride | 25/05/2016 | Single Enantiomer | 4 | C |
| **Strimvelis** | Severe Combined Immunodeficiency | autologous cd34+ enriched cell fraction that contains cd34+ cells transduced with retroviral vector that encodes for the human adenosine deaminase (ada) cdna sequence from human haematopoietic stem/progenitor (cd34+) cells | 26/05/2016 | Biologic | - | - |
| **Zavicefta** | Pneumonia, Bacterial; Soft Tissue Infections; Pneumonia; Urinary Tract | **avibactam sodium**, ceftazidime pentahydrate | 23/06/2016 | Single Enantiomer | 3 | 1N, 2C |

| | | | | | | |
|---|---|---|---|---|---|---|
| | Infections; Gram-Negative Bacterial Infections | | | | | |
| **Ongentys** | Parkinson Disease | opicapone | 24/06/2016 | Achiral | - | - |
| **Epclusa** | Hepatitis C, Chronic | sofosbuvir, **velpatasvir** | 6/07/2016 | Single Enantiomer | 6 | C |
| **Zepatier** | Hepatitis C, Chronic | **elbasvir**, grazoprevir | 22/07/2016 | Single Enantiomer | 5 | C |
| **Zepatier** | Hepatitis C, Chronic | elbasvir, **grazoprevir** | 22/07/2016 | Single Enantiomer | 7 | C |
| **Cinqaero** | Asthma | reslizumab | 15/08/2016 | Biologic | - | - |
| **Zalmoxis** | Hematopoietic Stem Cell Transplantation; Graft vs Host Disease | allogeneic t cells genetically modified with a retroviral vector encoding for a truncated form of the human low affinity nerve growth factor receptor (δlngfr) and the herpes simplex i virus thymidine kinase (hsv-tk mut2) | 18/08/2016 | Biologic | - | - |
| **Truberzi** | Irritable Bowel Syndrome; Diarrhea | eluxadoline | 19/09/2016 | Single Enantiomer | 2 | C |
| **Ibrance** | Breast Neoplasms | palbociclib | 9/11/2016 | Achiral | - | - |
| **Lartruvo** | Sarcoma | olaratumab | 9/11/2016 | Biologic | - | - |
| **Parsabiv** | Hyperparathyroidism, Secondary | etelcalcetide hydrochloride | 11/11/2016 | Biologic | - | - |
| **Ninlaro** | Multiple Myeloma | ixazomib citrate | 21/11/2016 | Single Enantiomer | 1 | C |
| **Venclyxt** | Leukemia, Lymphocytic, Chronic, B-Cell | venetoclax | 4/12/2016 | Achiral | - | - |
| **Ocaliva** | Liver Cirrhosis, Biliary | obeticholic acid | 12/12/2016 | Single Enantiomer | 9 | C |
| **Afstyla** | Hemophilia A | lonoctocog alfa | 4/01/2017 | Biologic | - | - |
| **Zinplava** | Enterocolitis, Pseudomembranous | bezlotoxumab | 18/01/2017 | Biologic | - | - |
| **Olumiant** | Arthritis, Rheumatoid | baricitinib | 13/02/2017 | Achiral | - | - |

| | | | | | | |
|---|---|---|---|---|---|---|
| **Alecensa** | Carcinoma, Non-Small-Cell Lung | alectinib hydrochloride | 16/02/2017 | Achiral | - | - |
| **Xeljanz** | Arthritis, Rheumatoid | tofacitinib | 22/03/2017 | Single Enantiomer | 2 | C |
| **Varuby** | Vomiting; Nausea; Cancer | rolapitant | 19/04/2017 | Single Enantiomer | 3 | C |
| **Axumin** | Prostatic Neoplasms; Radionuclide Imaging | fluciclovine (18f) | 21/05/2017 | Achiral | - | - |
| **Trumenba** | Meningitis, Meningococcal | neisseria meningitidis serogroup b fhbp (recombinant lipidated fhbp (factor h binding protein)) subfamily a; neisseria meningitidis serogroup b fhbp (recombinant lipidated fhbp (factor h binding protein)) subfamily b | 24/05/2017 | Biologic | - | - |
| **Brineura** | Neuronal Ceroid-Lipofuscinoses | cerliponase alfa | 30/05/2017 | Biologic | - | - |
| **Spinraza** | Muscular Atrophy, Spinal | nusinersen sodium | 30/05/2017 | Biologic | - | - |
| **Refixia** | Hemophilia B | nonacog beta pegol | 2/06/2017 | Biologic | - | - |
| **Kevzara** | Arthritis, Rheumatoid | sarilumab | 23/06/2017 | Biologic | - | - |
| **Besponsa** | Precursor Cell Lymphoblastic Leukemia-Lymphoma | inotuzumab ozogamicin | 28/06/2017 | Biologic | - | - |
| **Oxervate** | Keratitis | recombinant human nerve growth factor | 6/07/2017 | Biologic | - | - |
| **Spherox** | Cartilage Diseases | spheroids of human autologous matrix-associated chondrocytes | 10/07/2017 | Biologic | - | - |
| **Reagila** | Schizophrenia | cariprazine hydrochloride | 13/07/2017 | Achiral | - | - |
| **Kyntheum** | Psoriasis | brodalumab | 17/07/2017 | Biologic | - | - |
| **Veltassa** | Hyperkalemia | patiromer sorbitex calcium | 19/07/2017 | Polymer | - | - |
| **Maviret** | Hepatitis C, Chronic | **glecaprevir**, pibrentasvir | 26/07/2017 | Single Enantiomer | 7 | C |
| **Maviret** | Hepatitis C, Chronic | glecaprevir, **pibrentasvir** | 26/07/2017 | Single Enantiomer | 8 | C |
| **Vosevi** | Hepatitis C, Chronic | sofosbuvir, velpatasvir, **voxilaprevi** | 26/07/2017 | Single Enantiomer | 8 | C |

| | | | | | | |
|---|---|---|---|---|---|---|
| **Kisqali** | Breast Neoplasms | ribociclib succinate | 22/08/2017 | Achiral | - | - |
| **Fotivda** | Carcinoma, Renal Cell | tivozanib | 24/08/2017 | Achiral | - | - |
| **Xermelo** | Carcinoid Tumor; Neuroendocrine Tumors | telotristat etiprate | 17/09/2017 | Single Enantiomer | 2 | C |
| **Bavencio** | Neuroendocrine Tumors | avelumab | 18/09/2017 | Biologic | - | - |
| **Rydapt** | Leukemia, Myeloid, Acute; Mastocytosis | midostaurin | 18/09/2017 | Single Enantiomer | 4 | C |
| **Tecentriq** | Carcinoma, Transitional Cell; Carcinoma, Non-Small-Cell Lung; Urologic Neoplasms; Breast Neoplasms; Small Cell Lung Carcinoma | atezolizumab | 20/09/2017 | Biologic | - | - |
| **Lutathera** | Neuroendocrine Tumors | lutetium (177lu) oxodotreotide | 26/09/2017 | Biologic | - | - |
| **Dupixent** | Dermatitis, Atopic; Prurigo; Esophageal Diseases; Asthma; Sinusitis | dupilumab | 26/09/2017 | Biologic | - | - |
| **Tookad** | Prostatic Neoplasms | padeliporfin di-potassium | 10/11/2017 | Biologic | - | - |
| **Tremfya** | Psoriasis | guselkumab | 10/11/2017 | Biologic | - | - |
| **Zejula** | Fallopian Tube Neoplasms; Peritoneal Neoplasms; Ovarian Neoplasms | niraparib (tosilate monohydrate) | 16/11/2017 | Single Enantiomer | 1 | C |
| **Prevymis** | Cytomegalovirus Infections | letermovir | 8/01/2018 | Single Enantiomer | 1 | C |
| **Ocrevus** | Multiple Sclerosis | ocrelizumab | 8/01/2018 | Biologic | - | - |
| **Adynovi** | Hemophilia A | rurioctocog alfa pegol | 8/01/2018 | Biologic | - | - |
| **Fasenra** | Asthma | benralizumab | 8/01/2018 | Biologic | - | - |
| **Ozempic** | Diabetes Mellitus | semaglutide | 8/02/2018 | Biologic | - | - |
| **Crysvita** | Hypophosphatemia, Familial; Hypophosphatemic | burosumab | 19/02/2018 | Biologic | - | - |

| | | | | | | |
|---|---|---|---|---|---|---|
| | Rickets, X-Linked Dominant; Osteomalacia | | | | | |
| **Hemlibra** | Hemophilia A | emicizumab | 23/02/2018 | Biologic | - | - |
| **Steglatro** | Diabetes Mellitus, Type 2 | ertugliflozin l-pyroglutamic acid | 21/03/2018 | Single Enantiomer | 6 | C |
| **Shingrix** | Herpes Zoster | recombinant varicella zoster virus glycoprotein e | 21/03/2018 | Biologic | - | - |
| **Lokelma** | Hyperkalemia | sodium zirconium cyclosilicate | 22/03/2018 | Achiral | - | - |
| **Lamzede** | alpha-Mannosidosis | velmanase alfa | 23/03/2018 | Biologic | - | - |
| **Segluromet** | Diabetes Mellitus, Type 2 | **ertugliflozin l-pyroglutamic acid**, metformin hydrochloride | 23/03/2018 | Single Enantiomer | 6 | C |
| **Steglujan** | Diabetes Mellitus, Type 2 | **ertugliflozin l-pyroglutamic acid**, sitagliptin phosphate monohydrate | 23/03/2018 | Single Enantiomer | 6 | C |
| **Alofisel** | Rectal Fistula | darvadstrocel | 23/03/2018 | Biologic | - | - |
| **Alpivab** | Influenza, Human | peramivir | 13/04/2018 | Single Enantiomer | 5 | C |
| **Mylotarg** | Leukemia, Myeloid, Acute | gemtuzumab ozogamicin | 19/04/2018 | Biologic | - | - |
| **Rubraca** | Ovarian Neoplasms | rucaparib camsylate | 23/05/2018 | Single Enantiomer | 2 | C |
| **Biktarvy** | HIV Infections | **bictegravir**, emtricitabine, tenofovir alafenamide, fumarate | 21/06/2018 | Single Enantiomer | 3 | C |
| **Tegsedi** | Amyloidosis | inotersen sodium | 6/07/2018 | Biologic | - | - |
| **Aimovig** | Migraine Disorders | erenumab | 26/07/2018 | Biologic | - | - |
| **Rxulti** | Schizophrenia | brexpiprazole | 26/07/2018 | Achiral | - | - |
| **Myalepta** | Lipodystrophy, Familial Partial | metreleptin | 29/07/2018 | Biologic | - | - |
| **Kymriah** | Precursor B-Cell Lymphoblastic Leukemia-Lymphoma; Lymphoma, Large B-Cell, Diffuse | tisagenlecleucel | 22/08/2018 | Biologic | - | - |

| | | | | | | |
|---|---|---|---|---|---|---|
| **Yescarta** | Lymphoma, Follicular; Lymphoma, Large B-Cell, Diffuse | axicabtagene ciloleucel | 23/08/2018 | Biologic | - | - |
| **Mepsevii** | Mucopolysaccharidosis VII | vestronidase alfa | 23/08/2018 | Biologic | - | - |
| **Onpattro** | Amyloidosis, Familial | patisiran sodium | 27/08/2018 | Biologic | - | - |
| **Cablivi** | Purpura, Thrombotic Thrombocytopenic | caplacizumab | 30/08/2018 | Biologic | - | - |
| **Nerlynx** | Breast Neoplasms | neratinib | 31/08/2018 | Achiral | - | - |
| **Ilumetr** | Psoriasis | tildrakizumab | 17/09/2018 | Biologic | - | - |
| **Braftovi** | Melanoma; Colorectal Neoplasms | encorafenib | 19/09/2018 | Single Enantiomer | 1 | C |
| **Mektovi** | Melanoma | binimetinib | 20/09/2018 | Achiral | - | - |
| **Xerava** | Infection; Bacterial Infections | eravacycline | 20/09/2018 | Single Enantiomer | 4 | C |
| **Imfinzi** | Carcinoma, Non-Small-Cell Lung | durvalumab | 21/09/2018 | Biologic | - | - |
| **Verzenios** | Breast Neoplasms | abemaciclib | 26/09/2018 | Achiral | - | - |
| **Symkevi** | Cystic Fibrosis | tezacaftor, ivacaftor | 31/10/2018 | Achiral | - | - |
| **Emgality** | Migraine Disorders | galcanezumab | 14/11/2018 | Biologic | - | - |
| **Vabomere** | Urinary Tract Infections; Bacteremia; Bacterial Infections; Respiratory Tract Infections; Pneumonia; Pneumonia, Ventilator-Associated | meropenem trihydrate, **vaborbactam** | 20/11/2018 | Single Enantiomer | 2 | C |
| **Alunbrig** | Carcinoma, Non-Small-Cell Lung | brigatinib | 22/11/2018 | Achiral | - | - |
| **Poteligeo** | Sezary Syndrome; Mycosis Fungoides | mogamulizumab | 22/11/2018 | Biologic | - | - |
| **Delstrigo** | HIV Infections | **doravirine**, lamivudine, tenofovir disoproxil fumarate | 22/11/2018 | Achiral | - | - |

| | | | | | | |
|---|---|---|---|---|---|---|
| **Pifeltro** | HIV Infections | doravirine | 22/11/2018 | Achiral | - | - |
| **Jivi** | Hemophilia A | damoctocog alfa pegol | 22/11/2018 | Biologic | - | - |
| **Takhzyro** | Angioedemas, Hereditary | lanadelumab | 22/11/2018 | Biologic | - | - |
| **Luxturna** | Leber Congenital Amaurosis; Retinitis Pigmentosa | voretigene neparvovec | 22/11/2018 | Biologic | - | - |
| **Dengvaxia** | Dengue | chimeric yellow fever dengue virus serotype 1 (live, attenuated), chimeric yellow fever dengue virus serotype 2 (live, attenuated), chimeric yellow fever dengue virus serotype 3 (live, attenuated), chimeric yellow fever dengue virus serotype 4 (live, attenuated) | 12/12/2018 | Biologic | - | - |
| **GHRYVELIN (previously Macimorelin Aeterna Zentaris)** | Diagnostic Techniques, Endocrine | macimorelin acetate | 11/01/2019 | Single Enantiomer | 2 | C |
| **Erleada** | Prostatic Neoplasms | apalutamide | 14/01/2019 | Achiral | - | - |
| **Besremi** | Polycythemia Vera | ropeginterferon alfa-2b | 15/02/2019 | Biologic | - | - |
| **Mulpleo** | Thrombocytopenia | lusutrombopag | 18/02/2019 | Single Enantiomer | 1 | C |
| **Rizmoic** | Constipation | naldemedine tosilate | 18/02/2019 | Single Enantiomer | 4 | C |
| **Ajovy** | Migraine Disorders | fremanezumab | 28/03/2019 | Biologic | - | - |
| **Vizimpro** | Carcinoma, Non-Small-Cell Lung | dacomitinib monohydrate | 2/04/2019 | Achiral | - | - |
| **Zynquista** | Diabetes Mellitus, Type 1 | sotagliflozin | 26/04/2019 | Single Enantiomer | 5 | C |
| **Ondexxya** | Drug-Related Side Effects and Adverse Reactions | andexanet alfa | 26/04/2019 | Biologic | - | - |
| **Skyrizi** | Psoriasis; Arthritis, Psoriatic | risankizumab | 26/04/2019 | Biologic | - | - |
| **Palynziq** | Phenylketonurias | pegvaliase | 3/05/2019 | Biologic | - | - |
| **Waylivra** | Hyperlipoproteinemia Type I | volanesorsen sodium | 3/05/2019 | Biologic | - | - |

| | | | | | | |
|---|---|---|---|---|---|---|
| **Lorviqua** | Carcinoma, Non-Small-Cell Lung | lorlatinib | 6/05/2019 | Single Enantiomer | 1 | C |
| **Zynteglo** | beta-Thalassemia | autologous cd34+ cell enriched population that contains hematopoietic stem cells transduced with lentiglobin bb305 lentiviral vector encoding the beta-a-t87q-globin gene | 29/05/2019 | Biologic | - | - |
| **Talzenna** | Breast Neoplasms | talazoparib | 20/06/2019 | Single Enantiomer | 2 | C |
| **Doptelet** | Thrombocytopenia | avatrombopag maleate | 20/06/2019 | Achiral | - | - |
| **Esperoct** | Hemophilia A | turoctocog alfa pegol | 20/06/2019 | Biologic | - | - |
| **Libtayo** | Carcinoma, Squamous Cell | cemiplimab | 28/06/2019 | Biologic | - | - |
| **Ultomiris** | Hemoglobinuria, Paroxysmal | ravulizumab | 2/07/2019 | Biologic | - | - |
| **Giapreza** | Hypotension; Shock | angiotensin ii acetate | 23/08/2019 | Biologic | - | - |
| **Vitrakvi** | Abdominal Neoplasms | larotrectinib sulfate | 19/09/2019 | Single Enantiomer | 2 | C |
| **Trogarzo** | HIV Infections | ibalizumab | 26/09/2019 | Biologic | - | - |
| **Xospata** | Leukemia, Myeloid, Acute | gilteritinib fumarate | 24/10/2019 | Achiral | - | - |
| **Ervebo** | Hemorrhagic Fever, Ebola | recombinant vesicular stomatitis virus (strain indiana) with a deletion of the envelope glycoprotein, replaced with the zaire ebolavirus (strain kikwit 1995) surface glycoprotein | 11/11/2019 | Biologic | - | - |
| **Rhokiinsa** | Glaucoma, Open-Angle; Ocular Hypertension | netarsudil | 19/11/2019 | Single Enantiomer | 1 | C |
| **Evenity** | Osteoporosis | romosozumab | 9/12/2019 | Biologic | | |
| **Quofenix** | Community-Acquired Infections | delafloxacin meglumine | 16/12/2019 | Single Enantiomer | 4 | C |
| **Rinvoq** | Arthritis, Rheumatoid | upadacitinib | 16/12/2019 | Single Enantiomer | 2 | C |
| **Tavlesse** | Thrombocytopenia | fostamatinib disodium | 9/01/2020 | Achiral | - | - |
| **Isturisa** | Cushing Syndrome | osilodrostat phosphate | 9/01/2020 | Single Enantiomer | 1 | C |

| | | | | | | |
|---|---|---|---|---|---|---|
| **Mayzent** | Multiple Sclerosis, Relapsing-Remitting | siponimod fumaric acid | 13/01/2020 | Achiral | - | - |
| **Polivy** | Lymphoma, B-Cell | polatuzumab vedotin | 16/01/2020 | Biologic | - | - |
| **Sunosi** | Narcolepsy; Sleep Apnea, Obstructive | solriamfetol hydrochloride | 16/01/2020 | Single Enantiomer | 1 | C |
| **Recarbrio** | Gram-Negative Bacterial Infections | imipenem monohydrate, cilastatin sodium, relebactam monohydrate | 13/02/2020 | Single Enantiomer | 3 | C |
| **Beovu** | Wet Macular Degeneration | brolucizumab | 13/02/2020 | Biologic | - | - |
| **Givlaari** | Porphyrias, Hepatic | givosiran | 2/03/2020 | Biologic | - | - |
| **Nubeqa** | Prostatic Neoplasms, Castration-Resistant | darolutamide | 27/03/2020 | Biologic | - | - |
| **Nustendi** | Hypercholesterolemia; Dyslipidemias | bempedoic acid, ezetimibe | 27/03/2020 | Achiral | - | - |
| **Staquis** | Dermatitis, Atopic | crisaborole | 27/03/2020 | Achiral | - | - |
| **Vaxchora** | Cholera | vibrio cholerae, strain cvd 103-hgr, live | 1/04/2020 | Biologic | - | - |
| **Nilemdo** | Hypercholesterolemia; Dyslipidemias | bempedoic acid | 1/04/2020 | Achiral | - | - |
| **Zolgensma** | Muscular Atrophy, Spinal | onasemnogene abeparvovec | 18/05/2020 | Biologic | - | - |
| **Sarclisa** | Multiple Myeloma | isatuximab | 30/05/2020 | Biologic | - | - |
| **Daurismo** | Leukemia, Myeloid, Acute | glasdegib maleate | 26/06/2020 | Single Enantiomer | 2 | C |
| **Mvabea** | Hemorrhagic Fever, Ebola | recombinant modified vaccinia ankara bavarian nordic virus encoding the: ebola virus zaire (zebov) mayinga strain glycoprotein (gp); ebola virus sudan gulu strain gp; ebola virus taï forest strain nucleoprotein and the marburg virus musoke strain gp | 1/07/2020 | Biologic | - | - |
| **Zabdeno** | Hemorrhagic Fever, Ebola | recombinant adenovirus type 26 (ad26) encoding the glycoprotein (gp) of the ebola virus zaire (zebov) mayinga strain | 1/07/2020 | Biologic | - | - |
| **Veklury** | COVID-19 virus infection | remdesivir | 3/07/2020 | Single Enantiomer | 6 | 1 P, 5 C |

| | | | | | | |
|---|---|---|---|---|---|---|
| **Xenleta** | Pneumonia, Bacterial; Community-Acquired Infections | lefamulin acetate | 27/07/2020 | Single Enantiomer | 11 | C |
| **Rozlytrek** | Cancer; Carcinoma, Non-Small-Cell Lung | entrectinib | 31/07/2020 | Achiral | - | - |
| **Hepcludex** | Hepatitis D, Chronic | bulevirtide acetate | 31/07/2020 | Biologic | - | - |
| **Dovprela** | Tuberculosis, Multidrug-Resistant | pretomanid | 31/07/2020 | Single Enantiomer | 1 | C |
| **Kaftrio** | Cystic Fibrosis | ivacaftor, tezacaftor, elexacaftor | 21/08/2020 | Achiral | - | - |
| **Blenrep** | Multiple Myeloma | belantamab mafodotin | 25/08/2020 | Biologic | - | - |
| **Idefirix** | Desensitization, Immunologic; Kidney Transplantation | imlifidase | 25/08/2020 | Biologic | - | - |
| **Ayvakyt** | Gastrointestinal Stromal Tumors | avapritinib | 24/09/2020 | Single Enantiomer | 1 | C |
| **Jyseleca** | Arthritis, Rheumatoid | filgotinib maleate | 24/09/2020 | Achiral | - | - |
| **Adakveo** | Anemia, Sickle Cell | crizanlizumab | 28/10/2020 | Biologic | - | - |
| **Calquence** | Leukemia, Lymphocytic, Chronic, B-Cell | acalabrutinib | 5/11/2020 | Single Enantiomer | 1 | C |
| **Supemtek** | Influenza, Human | influenza a virus subtype h1n1 haemagglutinin, recombinant, influenza a virus subtype h3n2 haemagglutinin, recombinant, influenza b virus victoria lineage haemagglutinin, recombinant, influenza b virus yamagata lineage haemagglutinin, recombinant | 16/11/2020 | Biologic | - | - |
| **Nyxthracis (previously Obiltoxaximab SFL)** | Anthrax | nyxthracis | 18/11/2020 | Biologic | - | - |
| **Oxlumo** | Hyperoxaluria, Primary | lumasiran sodium | 19/11/2020 | Biologic | - | - |
| **Tecartus** | Lymphoma, Mantle-Cell | autologous peripheral blood t cells cd4 and cd8 selected and cd3 and cd28 activated transduced with retroviral vector expressing | 14/12/2020 | Biologic | - | - |

| | | | | | | |
|---|---|---|---|---|---|---|
| | | anti-cd19 cd28/cd3-zeta chimeric antigen receptor and cultured (brexucabtagene autoleucel) | | | | |
| **Vocabria** | HIV Infections | cabotegavir sodium, cabotegravir | 17/12/2020 | Single Enantiomer | 2 | C |
| **Libmeldy** | Leukodystrophy, Metachromatic | atidarsagene autotemcel | 17/12/2020 | Biologic | - | - |
| **Phesgo** | Breast Neoplasms | pertuzumab, trastuzumab | 21/12/2020 | Biologic | - | - |
| **Comirnaty** | COVID-19 virus infection | single-stranded, 5'-capped messenger rna produced using a cell-free in vitro transcription from the corresponding dna templates, encoding the viral spike (s) protein of sars-cov-2 | 21/12/2020 | Biologic | - | - |
| **Spikevax** | COVID-19 virus infection | cx-024414 (single-stranded, 5'-capped messenger rna (mrna) produced using a cell-free in vitro transcription from the corresponding dna templates, encoding the viral spike (s) protein of sars-cov-2) | 6/01/2021 | Biologic | - | - |
| **Elzonris** | Lymphoma | tagraxofusp | 7/01/2021 | Biologic | - | - |
| **Xofluza** | Influenza, Human | baloxavir marboxil | 7/01/2021 | Single Enantiomer | 2 | C |
| **Enhertu** | Breast Neoplasms | trastuzumab deruxtecan | 18/01/2021 | Biologic | - | - |
| **Vaxzevria** | COVID-19 virus infection | chadox1-sars-cov-2 | 29/01/2021 | Biologic | - | - |
| **Rukobia** | HIV Infections | fostemsavir trometamol | 4/02/2021 | Achiral | - | - |
| **Inrebic** | Myeloproliferative Disorders; Primary Myelofibrosis | fedratinib dihydrochloride monohydrate | 8/02/2021 | Achiral | - | - |
| **Lumoxiti** | Leukemia, Hairy Cell | moxetumomab pasudotox | 8/02/2021 | Biologic | - | - |
| **Retsevmo** | Carcinoma, Non-Small-Cell Lung; Thyroid Neoplasms | selpercatinib | 11/02/2021 | Achiral | - | - |
| **Tukysa** | Breast Neoplasms; Neoplasm Metastasis | tucatinib | 11/02/2021 | Achiral | - | - |
| **Jcovden** | COVID-19 virus infection | adenovirus type 26 encoding the sars-cov-2 spike glycoprotein (ad26.cov2-s) | 11/03/2021 | Biologic | - | - |
| **Nexpovio** | Multiple Myeloma | selinexor | 26/03/2021 | Achiral | - | - |

| | | | | | | |
|---|---|---|---|---|---|---|
| **Pemazyre** | Cholangiocarcinoma | pemigatinib | 26/03/2021 | Achiral | - | - |
| **Byfavo** | Conscious Sedation | remimazolam | 26/03/2021 | Single Enantiomer | 1 | C |
| **Evrysdi** | Muscular Atrophy, Spinal | risdiplam | 26/03/2021 | Achiral | - | - |
| **Vazkepa** | Dyslipidemias | icosapent ethyl | 26/03/2021 | Achiral | - | - |
| **Sogroya** | Growth | somapacitan | 31/03/2021 | Biologic | - | - |
| **Jemperli** | Endometrial Neoplasms | dostarlimab | 21/04/2021 | Biologic | - | - |
| **Copiktra** | Leukemia, Lymphocytic, Chronic, B-Cell; Lymphoma, Follicular | duvelisib | 19/05/2021 | Single Enantiomer | 1 | C |
| **Drovelis** | Contraceptives, Oral | drospirenone, **estetrol monohydrate** | 19/05/2021 | Single Enantiomer | 7 | C |
| **Lydisilka** | Contraceptives, Oral | **estetrol monohydrate**, drospirenone | 19/05/2021 | Single Enantiomer | 7 | C |
| **Koselugo** | Neurofibromatosis 1 | selumetinib sulfate | 17/06/2021 | Achiral | - | - |
| **Evkeeza** | Hypercholesterolemia | evinacumab | 17/06/2021 | Biologic | - | - |
| **Enspryng** | Neuromyelitis Optica | satralizumab | 24/06/2021 | Biologic | - | - |
| **Skysona** | Adrenoleukodystrophy | elivaldogene autotemcel | 16/07/2021 | Biologic | - | - |
| **Imcivree** | Obesity | setmelanotide | 16/07/2021 | Biologic | - | - |
| **Verquvo** | Heart Failure | vericiguat | 16/07/2021 | Achiral | - | - |
| **Klisyri** | Keratosis, Actinic | tirbanibulin | 16/07/2021 | Achiral | - | - |
| **Ryeqo** | Leiomyoma | **relugolix**, norethisterone acetate, estradiol hemihydrate | 16/07/2021 | Achiral | - | - |
| **Bylvay** | Cholestasis, Intrahepatic | odevixibat | 16/07/2021 | Single Enantiomer | 2 | C |
| **Abecma** | Multiple Myeloma; Neoplasms; Cancer; Neoplasms, Plasma Cell; Hemostatic Disorders; Vascular Diseases; Cardiovascular Diseases; | idecabtagene vicleucel | 18/08/2021 | Biologic | - | - |

| | | | | | | |
|---|---|---|---|---|---|---|
| | Paraproteinemias; Blood Protein Disorders; Hematologic Diseases; Hemic and Lymphatic Diseases; Hemorrhagic Disorders; Infectious Mononucleosis; Lymphoproliferative Disorders; Immunoproliferative Disorders; Immune System Diseases | | | | | |
| **Evrenzo** | Anemia; Kidney Failure, Chronic | roxadustat | 18/08/2021 | Achiral | - | - |
| **Bimzelx** | Psoriasis | bimekizumab | 20/08/2021 | Biologic | - | - |
| **Minjuvi** | Lymphoma, Large B-Cell, Diffuse | tafasitamab | 26/08/2021 | Biologic | - | - |
| **Voxzogo** | Achondroplasia | vosoritide | 26/08/2021 | Biologic | - | - |
| **Regkirona** | COVID-19 virus infection | regdanvimab | 12/11/2021 | Biologic | - | - |
| **Ronapreve** | COVID-19 virus infection | casirivimab, imdevimab | 12/11/2021 | Biologic | - | - |
| **Gavreto** | Carcinoma, Non-Small-Cell Lung | pralsetinib | 18/11/2021 | Single Enantiomer | 1 | C |
| **Qinlock** | Gastrointestinal Stromal Tumors | ripretinib | 18/11/2021 | Achiral | - | - |
| **Trodelvy** | Breast Neoplasms; Triple Negative Breast Neoplasms | sacituzumab govitecan | 22/11/2021 | Biological | - | - |
| **Brukinsa** | Waldenstrom Macroglobulinemia | zanubrutinib | 22/11/2021 | Single Enantiomer | 1 | C |
| **Artesunate Amivas** | Malaria | artesunate | 22/11/2021 | Single Enantiomer | 8 | C |

| | | | | | | |
|---|---|---|---|---|---|---|
| **Rybrevant** | Carcinoma, Non-Small-Cell Lung | amivantamab | 9/12/2021 | Biologic | - | - |
| **Cibinqo** | Dermatitis, Atopic | abrocitinib | 9/12/2021 | Achiral | - | - |
| **Vaxneuvance** | Pneumococcal Infections | pneumococcal polysaccharide conjugate vaccine (adsorbed) | 13/12/2021 | Biologic | - | - |
| **Xevudy** | COVID-19 virus infection | sotrovimab | 17/12/2021 | Biologic | - | - |
| **Nuvaxovid** | COVID-19 virus infection | sars-cov-2 recombinant spike protein | 20/12/2021 | Biologic | - | - |
| **Lumykras** | Carcinoma, Non-Small-Cell Lung | sotorasib | 6/01/2022 | Single Enantiomer | 1 | C |
| **Tecovirimat SIGA** | Poxviridae Infections; Cowpox; Monkeypox; Vaccinia; Smallpox | tecovirimat | 6/01/2022 | Single Enantiomer | 6 | C |
| **Voraxaze** | Metabolic Side Effects of Drugs and Substances | glucarpidase | 11/01/2022 | Biologic | - | - |
| **Skytrofa** | Growth and Development | lonapegsomatropin | 11/01/2022 | Biologic | - | - |
| **Tavneos** | Microscopic Polyangiitis; Wegener Granulomatosis | avacopan | 11/01/2022 | Single Enantiomer | 2 | C |
| **Vyepti** | Migraine Disorders | eptinezumab | 24/01/2022 | Biologic | - | - |
| **Paxlovid** | COVID-19 virus infection | **nirmatrelvir**, ritonavir | 28/01/2022 | Single Enantiomer | 6 | C |
| **Ngenla** | Growth and Development | somatrogon | 14/02/2022 | Biologic | - | - |
| **Oxbryta** | Anemia; Anemia, Hemolytic; Anemia, Sickle Cell | voxelotor | 14/02/2022 | Achiral | - | - |
| **Saphnelo** | Lupus Erythematosus, Systemic | anifrolumab | 14/02/2022 | Biologic | - | - |
| **Apexxnar** | Pneumococcal Infections | pneumococcal polysaccharide serotype 1, pneumococcal polysaccharide serotype 3, pneumococcal polysaccharide serotype 4, pneumococcal polysaccharide serotype 5, pneumococcal polysaccharide serotype 6a, pneumococcal polysaccharide serotype 6b, pneumococcal polysaccharide serotype 7f, pneumococcal polysaccharide serotype 8, pneumococcal | 14/02/2022 | Biologic | - | - |

| | | | | | | |
|---|---|---|---|---|---|---|
| | | polysaccharide serotype 9v, pneumococcal polysaccharide serotype 10a, pneumococcal polysaccharide serotype 11a, pneumococcal polysaccharide serotype 12f, pneumococcal polysaccharide serotype 14, pneumococcal polysaccharide serotype 15b, pneumococcal polysaccharide serotype 18c, pneumococcal polysaccharide serotype 19a, pneumococcal polysaccharide serotype 19f, pneumococcal polysaccharide serotype 22f, pneumococcal polysaccharide serotype 23f, pneumococcal polysaccharide serotype 33f | | | | |
| **Tepmetko** | Carcinoma, Non-Small-Cell Lung | tepotinib hydrochloride monohydrate | 16/02/2022 | Achiral | - | - |
| **Kerendia** | Renal Insufficiency, Chronic; Diabetes Mellitus, Type 2 | finerenone | 16/02/2022 | Single Enantiomer | 1 | C |
| **Evusheld** | COVID-19 virus infection | tixagevimab, cilgavimab | 25/03/2022 | Biologic | - | - |
| **Kimmtrak** | Uveal Neoplasms | tebentafusp | 1/04/2022 | Biologic | - | - |
| **Breyanzi** | Lymphoma, Large B-Cell, Diffuse; Lymphoma, Follicular; Mediastinal Neoplasms | cd19-directed genetically modified autologous cell-based product consisting of purified cd8+ t-cells (cd8+ cells), cd19-directed genetically modified autologous cell-based product consisting of purified cd4+ t cells (cd4+ cells) | 4/04/2022 | Biologic | - | - |
| **Padcev** | Carcinoma, Transitional Cell; Urologic Neoplasms | enfortumab vedotin | 13/04/2022 | Biologic | - | - |
| **Vydura** | Migraine Disorders | rimegepant | 25/04/2022 | Single Enantiomer | 3 | C |
| **Kapruvia** | Pruritus | difelikefalin | 25/04/2022 | Biologic | - | - |
| **Uplizna** | Neuromyelitis Optica | inebilizumab | 25/04/2022 | Biologic | - | - |
| **Quviviq** | Sleep Initiation and Maintenance Disorders | daridorexant hydrochloride | 29/04/2022 | Single Enantiomer | 1 | C |
| **Carvykti** | Multiple Myeloma | ciltacabtagene autoleucel | 25/05/2022 | Biologic | - | - |
| **Lunsumio** | Lymphoma, Follicular | mosunetuzumab | 3/06/2022 | Biologic | - | - |
| **Yselty** | Leiomyoma | linzagolix choline | 14/06/2022 | Achiral | - | - |

| | | | | | | |
|---|---|---|---|---|---|---|
| **Tabrecta** | Carcinoma, Non-Small-Cell Lung | capmatinib dihydrochloride monohydrate | 20/06/2022 | Achiral | - | - |
| **Xenpozyme** | Acid sphingomyelinase deficiency (ASMD) type A/B or type B | olipudase alfa | 24/06/2022 | Biologic | - | - |
| **COVID-19 Vaccine Valneva** | COVID-19 virus infection | sars-cov-2 virus (inactivated) wuhan strain hcov-19 / italy / inmi1-isl / 2020 | 24/06/2022 | Biologic | - | - |
| **Upstaza** | Amino Acid Metabolism, Inborn Errors | eladocagene exuparvovec | 18/07/2022 | Biologic | | |
| **Zokinvy** | Progeria; Laminopathies | lonafarnib | 18/07/2022 | Single Enantiomer | 1 | C |
| **Vyvgart** | Myasthenia Gravis | efgartigimod alfa | 10/08/2022 | Biologic | - | - |
| **Rayvow** | Migraine Disorders | lasmiditan succinate | 17/08/2022 | Achiral | - | - |
| **Sunlenca** | HIV Infections | lenacapavir sodium | 17/08/2022 | Single Enantiomer | 3 | C |
| **Tecvayli** | Multiple Myeloma | teclistamab | 23/08/2022 | Biologic | - | - |
| **Roctavian** | Hemophilia A | valoctocogene roxaparvovec | 24/08/2022 | Biologic | - | - |
| **Opdualag** | Melanoma | nivolumab, relatlimab | 15/09/2022 | Biologic | - | - |
| **Amvuttra** | Amyloid Neuropathies, Familial | vutrisiran sodium | 15/09/2022 | Biologic | - | - |
| **Mounjaro** | Diabetes Mellitus, Type 2 | tirzepatide | 15/09/2022 | Biologic | - | - |
| **Nulibry** | Metal Metabolism, Inborn Errors | fosdenopterin hydrobromide dihydrate | 15/09/2022 | Single Enantiomer | 4 | C |
| **Lupkynis** | Lupus Nephritis | voclosporin | 15/09/2022 | Single Enantiomer | 12 | C |
| **Vabysmo** | Wet Macular Degeneration; Macular Edema; Diabetes Complications | faricimab | 15/09/2022 | Biologic | - | - |
| **Tezspire** | Asthma | tezepelumab | 19/09/2022 | Biologic | - | - |

| | | | | | | |
|---|---|---|---|---|---|---|
| **Beyfortus** | Respiratory syncytial virus | nirsevimab | 31/10/2022 | Biologic | - | - |
| **Pyrukynd** | Genetic Diseases, Inborn; Anemia, Hemolytic | mitapivat sulfate | 9/11/2022 | Achiral | - | - |
| **Livtencity** | Cytomegalovirus Infections | maribavir | 9/11/2022 | Single Enantiomer | 4 | C |
| **VidPrevtyn Beta** | COVID-19 virus infection | sars-cov-2 prefusion spike delta tm protein, recombinant (b.1.351 strain) | 10/11/2022 | Biologic | - | - |
| **Enjaymo** | Hemolysis; Anemia, Hemolytic, Autoimmune | sutimlimab | 15/11/2022 | Biologic | - | - |
| **Qdenga** | Dengue | dengue virus, serotype 2, expressing dengue virus, serotype 1, surface proteins, live, attenuated, dengue virus, serotype 2, expressing dengue virus, serotype 3, surface proteins, live, attenuated, dengue virus, serotype 2, expressing dengue virus, serotype 4, surface proteins, live, attenuated, dengue virus, serotype 2, live, attenuated | 5/12/2022 | Biologic | - | - |
| **Pluvicto** | Prostatic Neoplasms, Castration-Resistant | lutetium (177lu) vipivotide tetraxetan | 9/12/2022 | Biologic | - | - |
| **Spevigo** | Psoriasis | spesolimab | 9/12/2022 | Biologic | - | - |
| **Locametz** | Radionuclide Imaging | gozetotide | 9/12/2022 | Biologic | - | - |
| **Eladynos** | Osteoporosis, Postmenopausal; Osteoporosis | abaloparatide | 12/12/2022 | Biologic | - | - |
| **Ebvallo** | Lymphoproliferative Disorders | tabelecleucel | 16/12/2022 | Biologic | - | - |
| **Zynlonta** | Lymphoma, Large B-Cell, Diffuse; Lymphoma, B-Cell | loncastuximab tesirine | 20/12/2022 | Biologic | - | - |
| **Imjudo** | Carcinoma, Hepatocellular | tremelimumab | 20/02/2023 | Biologic | - | - |
| **Tremelimumab AstraZeneca** | Carcinoma, Non-Small-Cell Lung | tremelimumab | 20/02/2023 | Biologic | - | - |
| **Hemgenix** | Hemophilia B | etranacogene dezaparvovec | 20/02/2023 | Biologic | - | - |

## References


1. Moss GP. Basic terminology of stereochemistry (IUPAC Recommendations 1996). Pure and Applied Chemistry. 1996;68(12):2193-222.

2. European Medicines Agency [09 April 2023]. Available from: https://www.ema.europa.eu/en.

3. Download medicine data European Medicines Agency website: European Medicines Agency; 2023 [09 April 2023]. Available from: https://www.ema.europa.eu/en/medicines/download-medicine-data#european-public-assessment-reports-(epar)-section.

4. World Health Organisation. Guidance on the use of international nonproprietary names (INNs) for pharmaceutical substances 2017.

5. New Drugs at FDA: CDER's New Molecular Entities and New Therapeutic Biological Products 2023 [21 May 2023]. Available from: https://www.fda.gov/drugs/development-approval-process-drugs/new-drugs-fda-cders-new-molecular-entities-and-new-therapeutic-biological-products. .

6. PubChem, National Library of Medicine website: National Center for Biotechnology Information; [2023]. Available from: https://pubchem.ncbi.nlm.nih.gov/.

7. Global Substance Registration System [2023]. Available from: https://gsrs.ncats.nih.gov/ginas/app/beta/home.

8. Hancu G, Modroiu A. Chiral Switch: Between Therapeutical Benefit and Marketing Strategy. Pharmaceuticals. 2022;15(2):240.

9. Salsabili M, Caschetta C, Senne N, Schondelmeyer S. Naming of Chiral Drugs. Food and Drug Law Journal. 2020;75(1):65-87.

10. Agranat I, Wainschtein SR, Zusman EZ. The predicated demise of racemic new molecular entities is an exaggeration. Nature Reviews Drug Discovery. 2012;11(12):972-3.
